# The noncanonical role of the protease cathepsin D as a cofilin phosphatase

**DOI:** 10.1038/s41422-020-00454-w

**Published:** 2021-01-29

**Authors:** Yi-Jun Liu, Ting Zhang, Sicong Chen, Daxiao Cheng, Cunjin Wu, Xingyue Wang, Duo Duan, Liya Zhu, Huifang Lou, Zhefeng Gong, Xiao-Dong Wang, Margaret S. Ho, Shumin Duan

**Affiliations:** 1 Department of Neurobiology and Department of Neurology of Second Affiliated Hospital, Zhejiang University School of Medicine, Hangzhou, Zhejiang 310009 China; 2 Research Units for Emotion and Emotion Disorders, NHC and CAMS Key Laboratory of Medical Neurobiology, MOE Frontier Science Center for Brain Research and Brain-Machine Integration, School of Brain Science and Brain Medicine, Zhejiang University, Hangzhou, Zhejiang 310058 China; 3 Clinical Research Center, The Second Affiliated Hospital, Zhejiang University School of Medicine, Hangzhou, Zhejiang 310009 China; 4 Department of Psychiatry, Sir Run Run Shaw Hospital, Zhejiang University School of Medicine, Hangzhou, Zhejiang 310016 China; 5 School of Life Science and Technology, ShanghaiTech University, Shanghai, 201210 China

**Keywords:** Actin, Cytokinesis

## Abstract

Cathepsin D (cathD) is traditionally regarded as a lysosomal protease that degrades substrates in acidic compartments. Here we report cathD plays an unconventional role as a cofilin phosphatase orchestrating actin remodeling. In neutral pH environments, the cathD precursor directly dephosphorylates and activates the actin-severing protein cofilin independent of its proteolytic activity, whereas mature cathD degrades cofilin in acidic pH conditions. During development, cathD complements the canonical cofilin phosphatase slingshot and regulates the morphogenesis of actin-based structures. Moreover, suppression of cathD phosphatase activity leads to defective actin organization and cytokinesis failure. Our findings identify cathD as a dual-function molecule, whose functional switch is regulated by environmental pH and its maturation state, and reveal a novel regulatory role of cathD in actin-based cellular processes.

## Introduction

Cathepsin D (cathD) is a ubiquitous lysosomal aspartyl protease widely expressed in eukaryotic cells. Depending on the acidic pH-sensitive protonation of two critical aspartic residues, cathD cleaves various substrates in lysosomal compartments, thereby regulating a variety of physiological and pathological processes.^1^ However, emerging evidence has revealed potential roles of cathD outside lysosomes^2–4^ in modulating cellular processes such as apoptosis, cell cycle and autophagy after translocation to the cytosol and other subcellular compartments beyond optimal pH range of its proteolytic activity.^2,5–7^ Moreover, proteolytically inactive cathD promotes cell proliferation, tumorigenesis and tumor invasion as potently as wild-type cathD.^8–11^ These findings suggest an unknown non-canonical role of cathD independent of its traditional degradation properties.

The protein level of cathD is closely associated with actin-based cellular processes, such as cell motility and proliferation. In most motile immune cells, cathD is constantly expressed at high levels.^12,13^ Increased cathD level enhances the proliferation and invasion of cancer cells, which is positively correlated with poor prognosis.^14–16^ We recently found that cathD interacted with the actin-severing factor cofilin and regulated actin-based fast cell migration after translocation to cytosol.^17^ Intriguingly, although cofilin is widely expressed, only two specific cofilin phosphatases, chronophin (CIN) and slingshot (ssh), have been identified so far. CIN only exists in vertebrates, while ssh is predominantly expressed in epithelial tissues and absent in several species.^18^ Therefore, other cofilin phosphatases likely exist to control cofilin activity.^19^ Based on these findings, we speculated that cathD might coordinate actin remodeling by direct regulation of cofilin activity in neutral pH environments.

In the present study, we provide evidence that cathD regulates actin-remodeling by direct dephosphorylation of cofilin independent of acidic pH or its proteolytic activity. Downregulation of cathD level or mutation in cathD disrupts the development of actin-based structures and impairs mitosis and cytokinesis. Our study identifies cathD as a dual-function molecule under different pH conditions, and provides mechanistic insights into cathD-mediated biological processes such as actin-based structure development, mitosis, and cytokinesis.

## Results

### CathD regulates organization of actin-based extensions in vivo

**Fig. 1:**
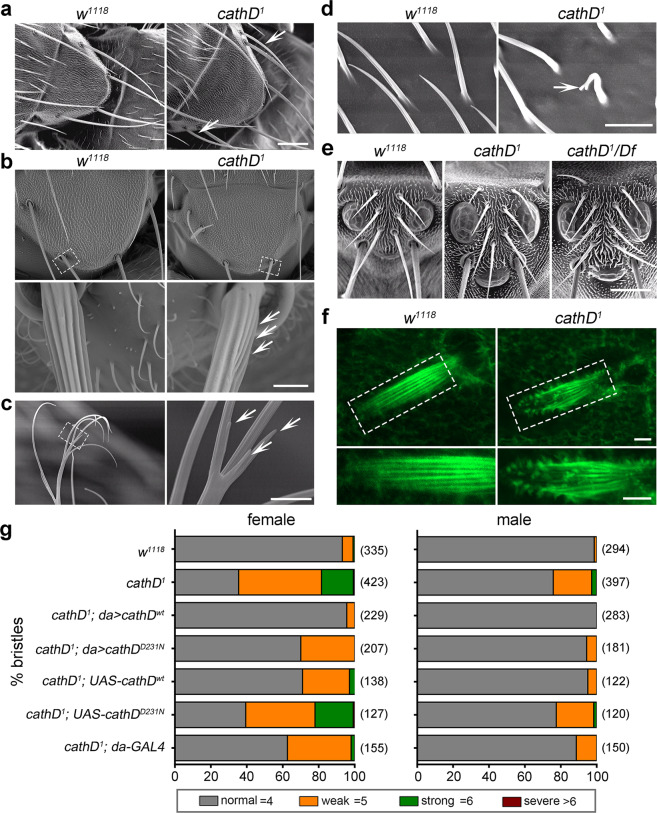
Loss of cathD leads to morphological defects in actin-based sensory organs. **a**–**e** Representative scanning electron microscope (SEM) images reveal morphological defects of actin-based sensory organs (indicated by arrows) in *cathD*^*1*^ mutant flies, including: ectopic bristles on the scutellum (**a**), disrupted actin organization on the bristle surface (**b**), aberrant split ends of antenna (**c**), microchaete with split tips (**d**), and ectopic ocellar bristles (**e**). Homozygous *cathD*^*1*^ mutants (**a**–**e**) and hemizygous mutants carrying *Df(2R)CA53* that deletes the entire *cathD* gene region on the second chromosome (**e**) were examined. **f** Representative images of notum bristles stained for F-actin (red) at 34 h after pupal formation (APF). *cathD*^*1*^ bristles exhibit disorganized microvillar-like short protrusions at the tips, whereas the wild-type bristles consist of organized parallel actin bundles. Lower panels, magnified views of dashed areas. **g** Scutellar bristles in adult flies were classified as follows based on severity: normal (gray, 4 bristles per scutellum), weak (orange, 5 bristles per scutellum), strong (green, 6 bristles per scutellum), and severe (dark red, > 6 bristles per scutellum). *n* = 120–423 flies per genotype from five independent experiments. *cathD*^*1*^ flies exhibit a significantly higher percentage of ectopic bristle number in both males and females, which could be restored by ubiquitous expression of wild-type human *cathD* (*cathD*^*1*^*; da* > *cathD*^*wt*^) or proteolytically inactive *cathD* (*cathD*^*1*^*; da* > *cathD*^*D231N*^). Scale bars, 50 μm for **a**, **e**, 10 μm for **b**, and 5 μm for **c**, **d**, **f**.

Actin cytoskeleton dynamics is tightly coordinated by various key factors during development.^20^ Recently, we identified the late endosome-derived cathD as a novel modulator of actin remodeling after its cytosolic translocation.^17^ CathD is evolutionarily conserved with a high homology from *Drosophila* to humans.^21^ To reveal whether cathD regulates actin organization in vivo, we examined actin-based sensory organs of *Drosophila*. Compared with wild-type controls (*w*^*1118*^), homozygous cathD-depleted flies (*cathD*^*1*^) exhibited developmental defects in actin-based structures, including extra scutellar bristles, macrochaete malformations, over-branched antenna, forked sensory hairs, and excess ocellar bristles (Fig. 1a–e). Visualized by fluorescent phalloidin staining, massive short protrusions or microvillar-like structures were observed at tips of pupal bristles in *cathD*^*1*^ mutant files, suggesting defects in actin patterning in the pupal stage (Fig. 1f).

As dysregulated actin dynamics promote the formation of ectopic bristles,^22–25^ scutellar bristles of homozygous *cathD*^*1*^ mutants were further analyzed to evaluate the developmental defects in actin-based structures. Compared with the wild-type controls, loss of cathD increased the number of ectopic bristles by ~10 fold (Fig. 1g). Driven by the *Daughterless* (*da*) promoter, ubiquitous expression of wild-type human cathD (cathD^wt^) completely abrogated ectopic bristle formation in cathD-depleted flies, whereas expressing the proteolytically inactive human cathD^D231N^ mutant also suppressed the formation of ectopic bristles (Fig. 1g), suggesting that cathD is necessary for the development of actin-based structures independent of its proteolytic activity.

### CathD directly dephosphorylates cofilin

**Fig. 2:**
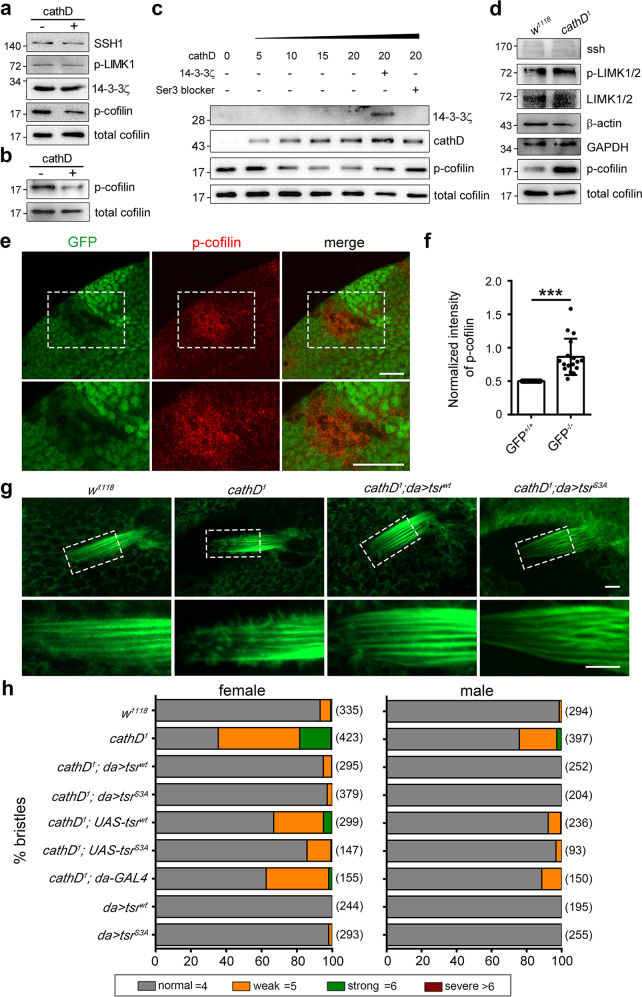
CathD dephosphorylates cofilin both in vitro and in vivo. **a** In vitro phosphatase assays showing that recombinant human cathD reduces phosphorylation levels of cofilin at Ser3 in whole-cell extracts from primary microglia. Levels of SSH1, 14-3-3ζ, p-LIMK, or total cofilin are comparable between groups (three independent experiments were performed). **b** In vitro phosphatase assays showing that recombinant human cathD exhibits phosphatase activity on purified human cofilin (1 h at 37 °C; five independent experiments were performed). **c** Recombinant cathD (5, 10, 15, and 20 nM) dephosphorylates purified cofilin (50 nM) in a dosage-dependent manner. Addition of recombinant 14-3-3ζ or Ser3-blocking peptide blocks cathD-mediated cofilin dephosphorylation (three independent experiments were performed). **d** Representative immunoblot images of total proteins extracted from wild-type (*w*^*1118*^) or cathD-depleted (*cathD*^*1*^) adult flies. p-cofilin levels are increased in *cathD*^*1*^ mutants. Three independent experiments were performed. **e, f** *cathD*^*1*^ mutant clones (GFP negative) were generated using FRT recombination in the 3rd instar larval wing discs. Representative images (**e**) and quantification (**f**) showing that p-cofilin levels are increased in *cathD*^*1*^mutant clones (GFP negative), compared with adjacent twinspot wild-type clones (GFP positive). *n* = 16 flies per group. Scale bar, 20 μm. **g** Representative images of actin-based fly bristles labeled with rhodamine-conjugated phalloidin. Dashed boxes are magnified in lower panels. Note that ubiquitous expression of either *tsr*^*wt*^ or *tsr*^*S3A*^ in the *cathD*^*1*^ mutant background (*cathD*^*1*^*; da* > *tsr*^*wt*^ or *cathD*^*1*^*; DA* > *tsr*^*S3A*^) rescues actin bristle defects in *cathD*^*1*^ mutants at 34 h APF. Scale bar, 5 μm. **h** Scutellar bristles in adult flies were classified as follows based on severity: normal (gray, 4 bristles per scutellum), weak (orange, 5 bristles per scutellum), strong (green, 6 bristles per scutellum), and severe (dark red, > 6 bristles per scutellum). *n* = 93–423 flies per genotype from six independent experiments. Ubiquitous expression of wild-type *tsr*^*wt*^ (*cathD*^*1*^*; da* > *tsr*^*wt*^) or constitutively active *tsr*^*S3A*^ (*cathD*^*1*^*; da* > *tsr*^*S3A*^) restores the bristle duplication phenotype in *cathD*^*1*^ flies. Data are means ± SEM, unpaired student *t-*test. ***P < 0.001. See Supplementary information, Table S3 for detailed statistics.

CathD shows a similar protein structure to the well-documented cofilin phosphatase CIN (Supplementary information, Fig. S1a), featuring a substrate-binding groove with paired aspartic residues responsible for nucleophilic attacks and group transfer reactions.^1,18^ This similarity drove us to ask whether cathD could directly activate cofilin as a CIN-like phosphatase in neutral pH environment. Therefore, we performed in vitro phosphatase assay using recombinant human cathD to interact with purified cofilin or whole-cell extracts. Our results showed that cathD directly decreased the phosphorylation level of cofilin in neutral pH buffer without affecting total cofilin or other regulatory elements (Fig. 2a, b), in a concentration-dependent manner (Fig. 2c). Furthermore, addition of recombinant scaffold protein 14-3-3ζ or exogenous Ser3-blocking peptide, which specifically binds with cofilin and protects the Ser3 residue from phosphatase attack,^26^ attenuated cathD-mediated decrease in phosphorylated cofilin (p-cofilin) levels (Fig. 2c). These results suggest that cathD exhibits cofilin phosphatase activities in neutral pH, under which cytosolic cathD likely executes its cofilin phosphatase function to mediate actin remodeling.

To unravel the cofilin-regulating role of cathD in vivo, we next detected the phosphorylation level of the *Drosophila* cofilin homolog *twinstar (tsr)* in cathD-depleted flies. Interestingly, *Drosophila* cofilin phosphorylation level was upregulated in the absence of cathD, while the total amount of cofilin and its regulatory factors were not affected (Fig. 2d). Loss of cathD also dramatically enhanced the fluorescence intensity of phosphorylated cofilin in mutant clones of larval wing discs (Fig. 2e, f). Furthermore, ubiquitous expression of wild-type cofilin (*tsr*^*wt*^) or its constitutively active mutant (*tsr*^*S3A*^) in *cathD*^*-/-*^ mutants reversed the malformation of microvillar-like protrusions in pupal bristles as well as the number of ectopic bristles in adults (Fig. 2g, h; Supplementary information, Fig. S1b), indicating that the loss of cathD induces actin-cytoskeleton malformations by suppressing the reactivation of endogenous cofilin. In addition, protein levels of total cofilin, its regulatory elements and F-actin were not significantly changed upon cathD-depletion (Supplementary information, Fig. S2a–h), implicating a selective regulatory role for cathD in processes requiring dynamic actin rearrangement such as bristle formation. These results suggest that cathD modulates actin arrangement pattern by dephosphorylating cofilin in vivo.

### CathD exhibits ssh-like phosphatase activity in cofilin activation

Currently, only one cofilin phosphatase, ssh, has been identified in *Drosophila* so far. Similar to *cathD*^*1*^ mutants, *ssh* null alleles (*ssh*^*1–63*^) homozygous mutant flies exhibited developmental defects in actin-based structures. These included abnormally aggregated actin-based structures in pupal bristles, extra scutellar bristles, over-branched antenna, and malformations of bristle surface (Supplementary information, Fig. S3a–d), besides bifurcated hairs reported previously.^27^ Intriguingly, ubiquitous expression of cathD^wt^ or proteolytically inactive cathD^D231N^ in *ssh*^*1–63*^ mutants reversed the phenotype of ectopic bristles, whereas *cathD*^*1*^ and *ssh*^*1–63*^ trans-heterozygotes synergistically aggravated bristle defects compared to heterozygous *cathD*^*1*^ or *ssh*^*1–63*^ mutants (Supplementary information, Fig. S3e). These data indicate that cathD acts similarly to ssh in actin regulation.

**Fig. 3:**
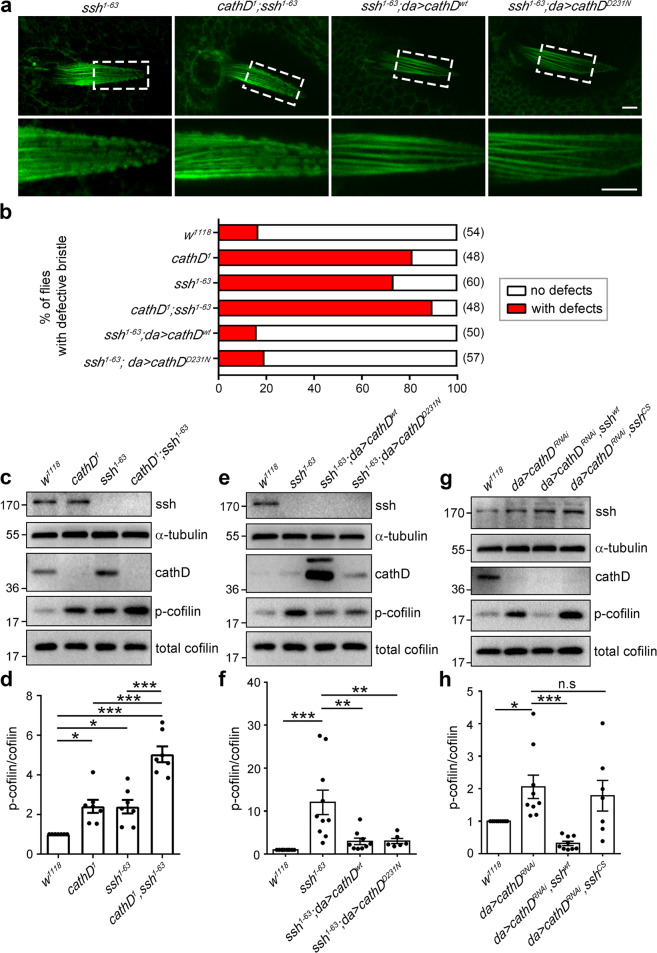
CathD exhibits slingshot-like phosphatase activity in cofilin activation. Representative staining images (**a**) and quantification (**b**) of F-actin-labeled pupal bristles. Note that ubiquitous expression of either cathD^wt^ or cathD^D231N^ in the *ssh*^*1–63*^ mutant background (*ssh*^*1–63*^*; da* > *cathD*^*wt*^ or *ssh*^*1–63*^*; da* > *cathD*^*D231N*^) reverses actin bristle defects in *ssh*^*1–63*^mutants, whereas double mutant of *cathD* and *ssh* (*cathD*^*1*^*; ssh*^*1–63*^) slightly aggravates the actin defects at 34 h APF. Dashed boxes are magnified in lower panels. Scale bar, 5 μm. Analysis of actin-based bristle structures in adult flies carrying different genotypes. Representative immunoblot images (**c**, **e**) and quantifications (**d**, **f**) of p-cofilin/cofilin ratio (normalized to wild-type controls*, w*^*1118*^) of total protein extracted from *ssh*^*1–63*^ mutant flies, showing elevated p-cofilin levels in double mutant of *cathD* and *ssh* (*cathD*^1^*; ssh*^*1–63*^), compared with *cathD*^*1*^ or *ssh*^*1–63*^(**c**, **d**). Expression of wild-type cathD (*ssh*^*1–63*^*; da* > *cathD*^*wt*^) or its proteolytically inactive mutant cathD^D231N^ (*ssh*^*1–63*^*; da* > *cathD*^*D231N*^) restores cofilin hyperphosphorylation in *ssh*^*1–63*^ (**e**, **f**). Representative immunoblot images (**g**) and quantifications (**h**) of total protein extracted from *da* > *cathD*^*RNAi*^ adult flies, showing that reducing cathD expression by *cathD* RNAi (*da* > *cathD*^*RNAi*^) increases p-cofilin levels. Co-expression of wild-type ssh effectively restores p-cofilin levels (*da* > *cathD*^*RNAi*^*, ssh*^*wt*^ flies), whereas ssh inactive mutation shows no effect (*da* > *cathD*^*RNAi*^*, ssh*^*CS*^). Data are means ± SEM. One-way ANOVA with Tukey’s post hoc test, *P < 0.05, **P < 0.01, ***P < 0.001.

Since double mutant of *cathD* and *ssh* (*cathD*^*1*^*; ssh*^*1–63*^) is lethal and all mutants die before eclosion, we next determined their actin phenotypes and the phosphorylation level of cofilin in the pupal stage. In our observation, loss of cathD increased malformation of microvillar-like protrusions on bristles and elevated p-cofilin level in *ssh*^*1–63*^pupae (Fig. 3a–d), which could be reversed by ubiquitous expression of cathD^wt^ or proteolytically inactive cathD^D231N^ (Fig. 3e, f). Consistent results were obtained when replacing *ssh*^*1–63*^ mutations with *ssh* RNAi, showing that elevated p-cofilin levels by ubiquitous expression of *ssh* RNAi was rescued by co-expression of either cathD or cathD^D231N^ (Supplementary information, Fig. S3f, g). Conversely, reducing cathD expression by expressing *cathD* RNAi led to cofilin hyperphosphorylation, which was effectively suppressed by co-expression of wild-type ssh (ssh^wt^). Co-expression of the catalytically inactive mutant ssh^cs^ also lowered p-cofilin levels but was less efficient (Fig. 3g, h). Taken together, cathD exerts ssh-like roles and serves as a novel cofilin phosphatase to regulate the development of actin-based structures.

### The cofilin phosphatase activity of cathD relies on pH conditions and its maturation state

**Fig. 4:**
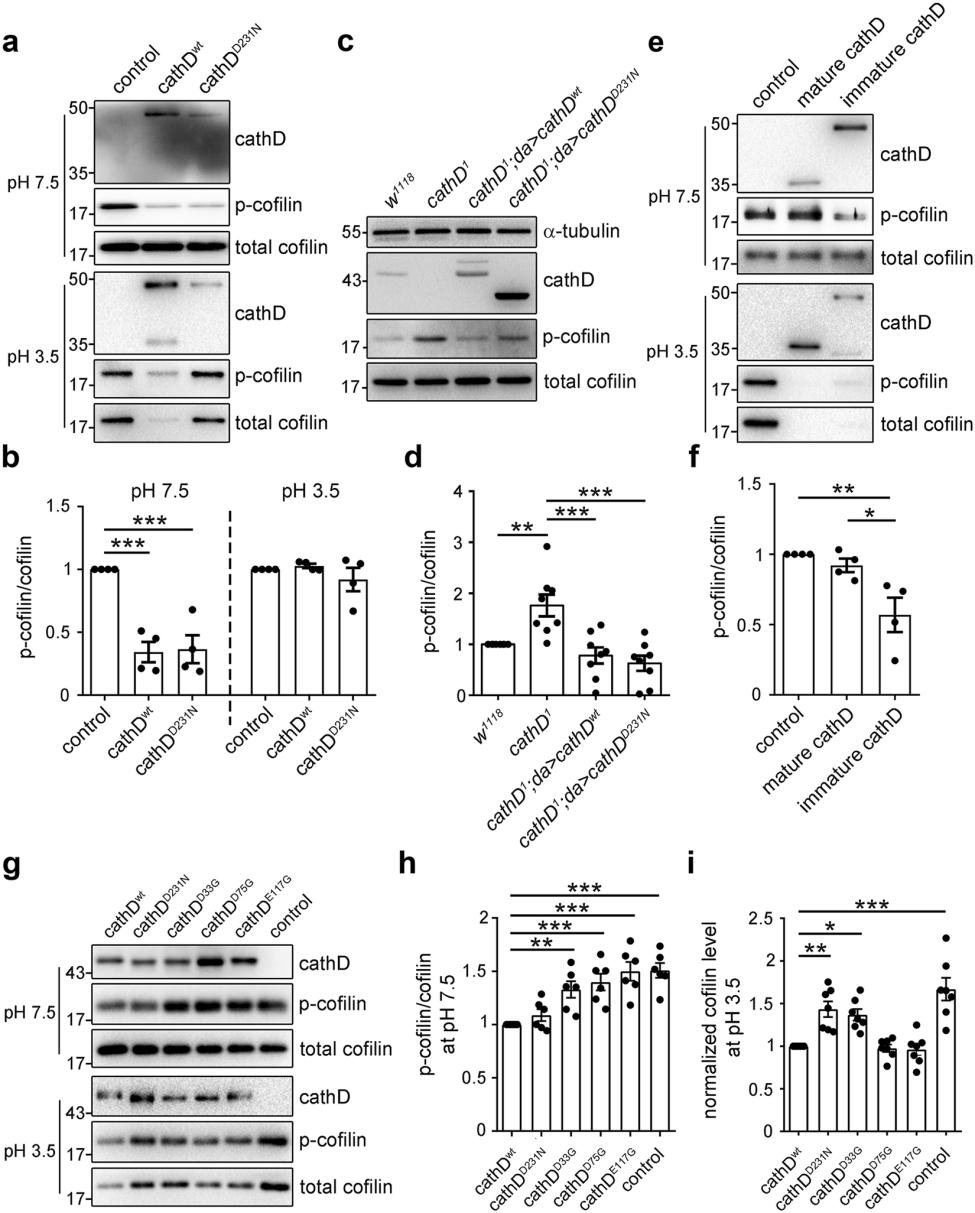
Functional switch of cathD activities relies on pH and its maturation. Representative immunoblot images (**a**) and quantification (**b**) of cathD-mediated cofilin phosphorylation in different pH conditions, showing both recombinant cathD^wt^ and cathD^D231N^ (10 nM) dephosphorylate cofilin in neutral pH (7.5), whereas cathD^wt^ mainly degrades cofilin in acidic pH (3.5). Note that both cathD^wt^ or cathD^D231N^ significantly reduce p-cofilin/cofilin ratio in neutral but not acidic pH (**c**). Representative immunoblot images (**c**) and quantification (**d**) of cathD-mediated cofilin phosphorylation in flies. **c** Elevated p-cofilin levels in *cathD*^*1*^ mutants are restored upon expressing either wild-type (*cathD*^*1*^*; da* > *cathD*^*wt*^) or proteolytically inactive cathD (*cathD*^*1*^*; da* > *cathD*^*D231N*^). **d** Quantification of p-cofilin/total cofilin levels (normalized to *w*^*1118*^ controls). The ratio is reduced when cathD^wt^ or cathD^D231N^ is expressed in *cathD*^*1*^ mutant background. Representative immunoblot images (**e**) and quantification (**f**) of in vitro phosphatase assay showing that immature recombinant human cathD directly dephosphorylates cofilin at pH 7.5, whereas mature cathD degrades cofilin at pH 3.5 (**e**). **f** Quantifications of p-cofilin/cofilin ratio (normalized to control) show that immature cathD dephosphorylates cofilin, whereas mature cathD shows limited phosphatase activity in neutral pH (7.5). Representative immunoblot images (**g**), quantification of p-cofilin/cofilin ratio (normalized to cathD^wt^, **h**) and cofilin level (normalized to cathD^wt^, **i**) from in vitro phosphatase assays using recombinant cathD^wt^, cathD^D231N^, cathD^D33G^, cathD^D75G^, and cathD^E117^. Note that cathD^D33G^, cathD^D75G^ and cathD^E117G^ exhibit impaired activities in cofilin dephosphorylation at pH 7.5, whereas cathD^D33G^ and cathD^D231N^ present suppressed proteolytic activities for cofilin degradation at pH 3.5. Data are means ± SEM, *P < 0.05, **P < 0.01, ***P < 0.001 by Mann–Whitney U test or One-way ANOVA with Tukey’s post hoc test.

To evaluate the catalytic property of cathD phosphatase activity, we examined the effects of general phosphatase inhibitors on the phosphatase activity of purified cathD. Similar to canonical cofilin phosphatases,^18,27^ cathD is insensitive to classical serine/threonine phosphatase inhibitors (Supplementary information, Fig. S4a, b). In a neutral pH environment (pH 7.5), both purified cathD^wt^ and its proteolytically inactive mutant cathD^D231N^ decreased p-cofilin levels without altering total cofilin protein level (Fig. 4a, b). These results indicate that the cofilin phosphatase activity of cathD in neutral pH is independent of its proteolytic activity. In comparison, in an acidic condition (pH 3.5), cathD^wt^ mainly played a hydrolytic role in cofilin degradation as shown by decreased total cofilin protein levels, whereas cathD^D231N^ showed impaired activity in cofilin degradation (Fig. 4a, b). Similarly, overexpression of cathD^wt^ or cathD^D231N^ restored the elevated p-cofilin levels in *cathD*^*1*^ mutants, suggesting that both forms exhibit cofilin phosphatase activities in vivo (Fig. 4c, d).

Since immature cathD is unstable and tends to yield the mature form by proteolysis in acidic pH conditions,^1^ the effects of immature and mature cathD on cofilin dephosphorylation in neutral and acidic pH were determined, respectively. We found that immature cathD directly dephosphorylated cofilin in neutral pH (Fig. 4e, f), whereas mature cathD mainly degraded cofilin in the acidic environment (Fig. 4e). These data suggest a dual role for cathD as both a ubiquitous protease and a cofilin phosphatase in different intracellular compartments, depending on environmental pH and its maturation state.

### Asp33, Asp75 and Glu117 residues are essential for cathD phosphatase activity

To elucidate the catalytic mechanism of cathD, candidate amino acid residues responsible for the phosphatase activity of cathD were screened by site-directed mutagenesis. Since charged acidic residues may activate nucleophilic attack for phosphate group transfer in catalysis,^28^ we speculated that aspartate (Asp) and glutamate (Glu) residues in the substrate-binding cleft might be involved. Therefore, nucleotide substitutions were made in amino acid positions 5, 12, 33, 50, 75, 117, 132, or 310 of human cathD, changing the residue Asp/Glu to Gly. Plasmids expressing cathD with different mutations were fused with a 6× His tag at the C-terminus and transfected to establish stable cell lines. Compared with the wild-type protein, cathD^D33G^, cathD^D75G^ or cathD^E117G^ mutants showed suppressed activities on cofilin dephosphorylation. In comparison, cathD^wt^, cath^D231N^ and other mutants markedly reduced the p-cofilin level (Supplementary information, Fig. S4c, d).

To verify the roles of these residues in cofilin regulation, we purified cathD mutant proteins without altering their stabilities, secondary structures, or binding abilities with cofilin (Supplementary information, Fig. S4e, f), and applied these proteins to in vitro phosphatase/protease assays. In our observation, purified cathD^D33G^, cathD^D75G^ and cathD^E117G^ exhibited impaired cofilin-phosphatase activities in a neutral pH environment (pH 7.5), whereas cathD^D33G^ and cathD^D231N^ showed suppressed proteolytic activities in an acidic condition (pH 3.5, Fig. 4g–i). We next reintroduced different cathD mutants into a cathD^-/-^ HeLa cell line, and examined the phosphorylation level of cofilin in these cells. We found that expression of cathD^wt^ or proteolytically inactive cathD^D231N^ dramatically reduced the p-cofilin level in cathD^-/-^ cells, whereas expression of phosphatase inactive cathD^D33G^, cathD^D75G^ or cathD^E117G^ mutants showed no effect (Supplementary information, Fig. S4g, h). These results suggest that Asp33, Asp75 and Glu117 are key residues for the phosphatase activity of cathD in a neutral pH environment, and Asp33 is essential for both phosphatase and proteolytic activities of cathD in both neutral and acidic pH environments.

**Fig. 5:**
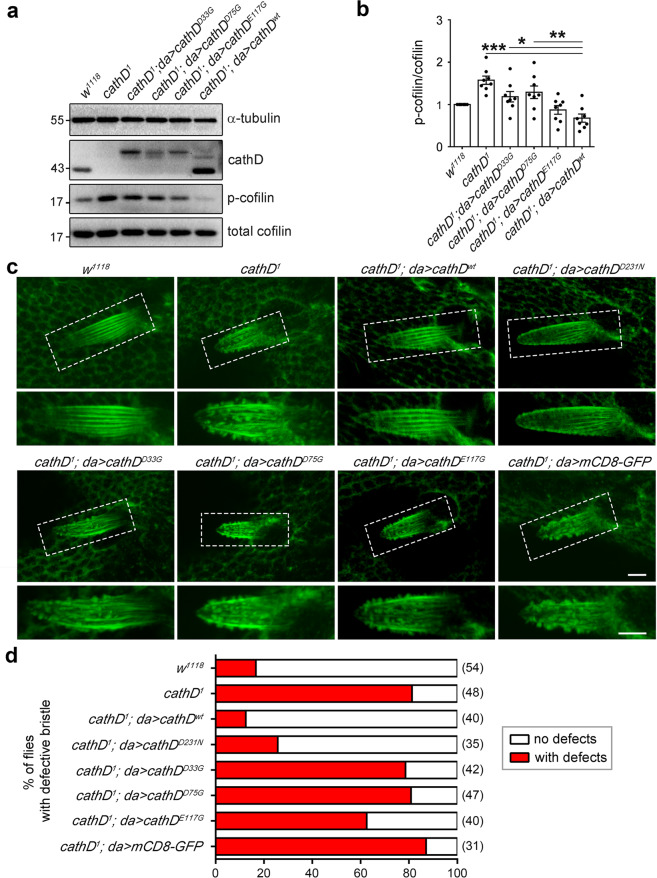
Asp33, Asp75, and Glu117 are required for the phosphatase activity of cathD. **a**, **b** Representative Western blots indicating p-cofilin levels in extracted proteins from adult flies. Expression of wild-type cathD fully rescues the elevated p-cofilin levels caused by cathD depletion (*cathD*^*1*^*; da* > *cathD*^wt^), whereas expression of mutant cathD carrying Asp33 (*cathD*^*1*^*; da* > *cathD*^D33G^), Asp75 (*cathD*^*1*^*; da* > *cathD*^D75G^), or Glu75 (*cathD*^*1*^*; da* > *cathD*^E117G^) in *cathD*^*1*^ background does not restore the elevated p-cofilin levels, suggesting that mutating any of these three residues abolishes cathD phosphatase activity. Quantifications of p-cofilin/cofilin ratio (normalized to *w*^*1118*^) are shown in **b**. Note that p-cofilin levels remain elevated upon expressing cathD mutants in *cathD*^*1*^ background (*cathD*^*1*^*; da* > *cathD*^D33G^, *cathD*^*1*^*; da* > *cathD*^D75G^ and *cathD*^*1*^*; da* > *cathD*^E117G^). **c** Actin-based bristle structures in pupae carrying different genotypes. Ubiquitous expression of the wild-type (*cathD*^*1*^*; da* > *cathD*^*wt*^) or a proteolytically inactive mutant of cathD (*cathD*^*1*^*; da* > *cathD*^*D231N*^) restores the over-protruded bristle phenotype upon cathD depletion (*cathD*^*1*^) at 34 h APF. No significant effects were detected upon expressing cathD^D33G^ (*cathD*^*1*^*; da* > *cathD*^D33G^), cathD^D75G^ (*cathD*^*1*^*; da* > *cathD*^D75G^) or cathD^E117G^ (*cathD*^*1*^*; da* > *cathD*^E117G^) and bristles remain defective. Bristles were labeled by phalloidin. Dash-boxed regions are enlarged in lower panels. **d** The ratio of individual flies with disorganized actin bundles in bristles, showing that ubiquitous expression of cathD^wt^ or a proteolytic inactive cathD^D231N^ reduces the defective ratio, whereas expression of cathD^D33G^, cathD^D75G^, or cathD^E117G^ exhibits minimal effect. Scale bar, 5 mm. Data are means ± SEM. One-way ANOVA with Tukey’s post hoc test, *P < 0.05, **P < 0.01, ***P < 0.001.

We further generated transgenic *Drosophila* lines ubiquitously expressing cathD^D33G^, cathD^D75G^ or cathD^E117G^ to examine the regulatory effect of these residues on cofilin activation. Compared with the wild-type cathD, mutation of Asp33, Asp75 or Glu117 suppressed dephosphorylation activity of cathD and attenuated cofilin-activation (Fig. 5a, b). Notably, cathD-mediated actin-cytoskeleton arrangement of *Drosophila* bristles and the percentage of flies with disordered F-actin bundles remained defective upon expressing cathD carrying any of these three mutations (Fig. 5c, d). In addition, expression of cathD^wt^ or cathD^D231N^ restored the elevated p-cofilin levels in LIMK1 overexpression larvae (Supplementary information, Fig. S5a, b), and reduced the lethality caused by LIMK1 overexpression, whereas cathD^D33G^, cathD^D75G^, or cathD^E117G^ had no effect (Supplementary information, Fig. S5c). Together, these results suggest that Asp33, Asp75, and Glu117 are essential residues for the phosphatase activity of cathD.

### CathD modulates mitosis and cytokinesis via its cofilin phosphatase activity

Cofilin is a pivotal modulator of cell mitosis.^18^ Perturbations of cofilin activation induce aberrant cytokinesis,^18,20^ and may lead to tumorigenesis with the accumulation of aneuploid cells.^29^ Notably, cathD has been shown to promote tumorigenesis and cancer growth independent of its proteolytic activity.^9,14,30^ Considering that immature cathD is its main form in cytosol (Supplementary information, Fig. S6a), whereas disrupting the expression level or phosphatase activity of cathD caused actin aggregation (Supplementary information, Fig. S6b, c), we speculated that cathD might modulate cell division via its phosphatase activity.

**Fig. 6:**
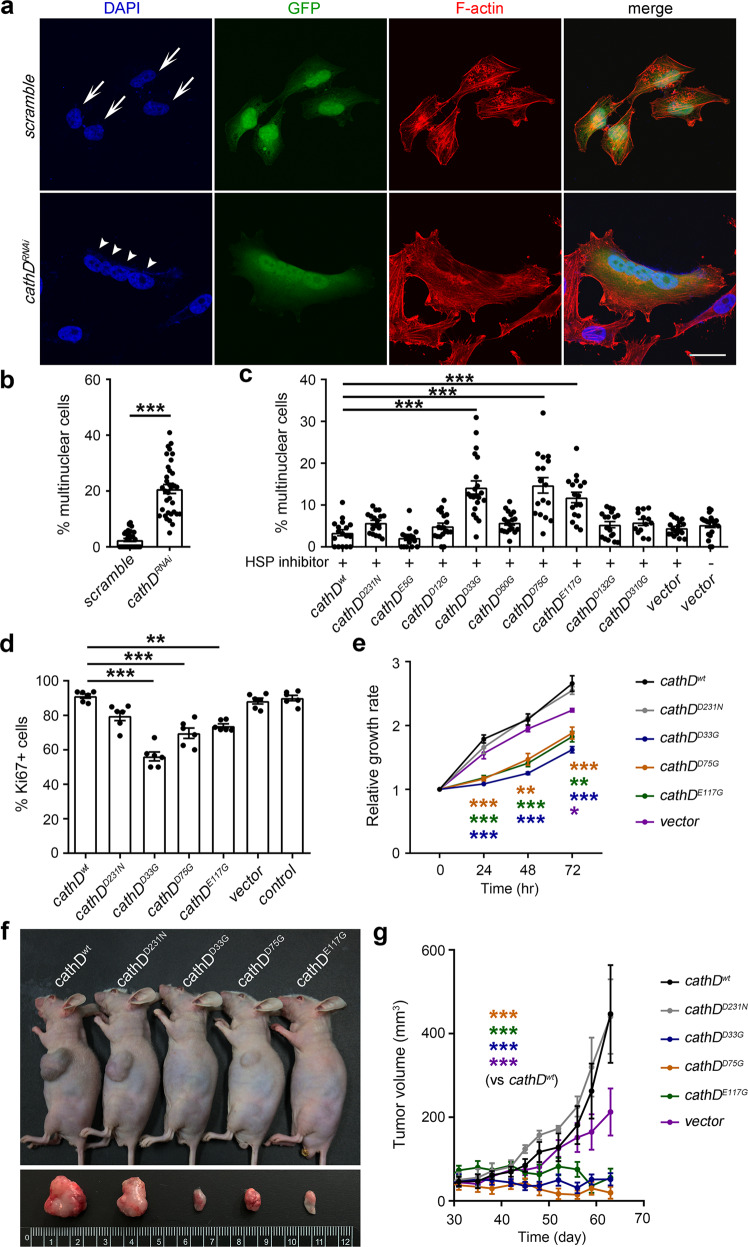
CathD regulates cell mitosis and cytokinesis via its cofilin phosphatase activity. **a**, **b** CathD-specific (*cathD*^*RNAi*^) or scrambled shRNA (*scramble*) was co-expressed with GFP in asynchronous HeLa cells. After cultured in 15 μM Hsp inhibitor I or vehicle for 48 h, cells were fixed and stained for F-actin with rhodamine-conjugated phalloidin (red). Nuclei were counter-stained with DAPI. (**a**) Representative results showing that downregulation of cathD by transfection of *cathD*-targeting shRNA generates giant multinuclear cells. Arrows indicate nuclei in control cells, while arrowheads indicate nuclei in a tetraploidy cell induced by *cathD* shRNA. Scale bar, 30 μm. (**b**) Quantifications of multinuclear HeLa cells. Compared with controls (*scramble*), downregulation of cathD (*cathD*^*RNAi*^) increases giant multinuclear cells. **c** A significant increase of giant multinuclear cells was found in cathD^D33G^, cathD^D75G^, and cathD^E117G^ mutants. Scale bar, 30 μm. **d** Quantifications of Ki67-positive A549 cells with indicated cathD mutants. Data are means ± SEM. One-way ANOVA followed with the Tukey’s test, **P < 0.01 and ***P < 0.001 compared with *cathD*^*wt*^ (*n* = 6). **e** CCK-8 assay of A549 cells at indicated time points. Relative proliferation ratios were normalized to the values at 0 h. **f** Representative images showing A549 xenografts carrying indicated cathD mutations in nude mice after subcutaneous transplantation. **g** The change in tumor volume of A549 xenografts (relative to the initial volume) carrying indicated cathD mutations (*n* = 9). Data are means ± SEM, *P < 0.05, **P < 0.01, and ***P < 0.001 the by Mann–Whitney U test, Kruskal–Wallis test (with Dunn’s test), or ANOVA (with Tukey’s test), respectively.

To verify our assumption, HeLa cells were transfected with plasmids expressing short hairpin RNA (shRNA) that targets *cathD*. Compared with the scrambled control, transfection of *cathD*-specific shRNA led to cytokinesis failure and the generation tetraploidy cells, indicating a dysfunction in cell mitosis and cytokinesis upon cathD depletion (Fig. 6a, b). We next generated stable cell lines expressing phosphatase-inactive mutants of cathD using the lentiviral system. Compared with controls, cathD^D33G^, cathD^D75G^ and cathD^E117G^ mutants significantly elevated the percentage of multinucleate cells with a high rate of aneuploidy (Fig. 6c; Supplementary information, Fig. S6d), suggesting that the regulatory role of cathD in cytokinesis depends on these residues.

Considering that cytokinesis defects lead to proliferative arrest,^29^ we further examined whether cathD could regulate cancer cell proliferation via its phosphatase activity. We chose lung cancer A549 cells that express low endogenous levels of cathD, and stably transfected these cells with either *cathD* or its mutants. Assessed by Ki67 staining and the CCK-8 proliferation assay, we found that overexpression of phosphatase-inactive cathD^D33G^, cathD^D75G^ and cathD^E117G^ mutants, rather than cathD^wt^ or proteolytically inactive cathD^D231N^, dramatically impaired proliferative activities of A549 cells (Fig. 6d, e), suggesting that the cathD mitogenicity relies on its phosphatase activity. Similar results were obtained in HeLa cells carrying phosphatase-inactive cathD mutants (Supplementary information, Fig. S6e–h). Furthermore, a remarkable suppression of tumor growth was observed in cathD^D33G^, cathD^D75G^ and cathD^E117G^ xenografts (Fig. 6f, g). Taken together, cathD requires Asp33, Asp75, and Glu117 residues to regulate cofilin activity and modulate cofilin-mediated processes such as cytokinesis and mitosis.

## Discussion

**Fig. 7:**
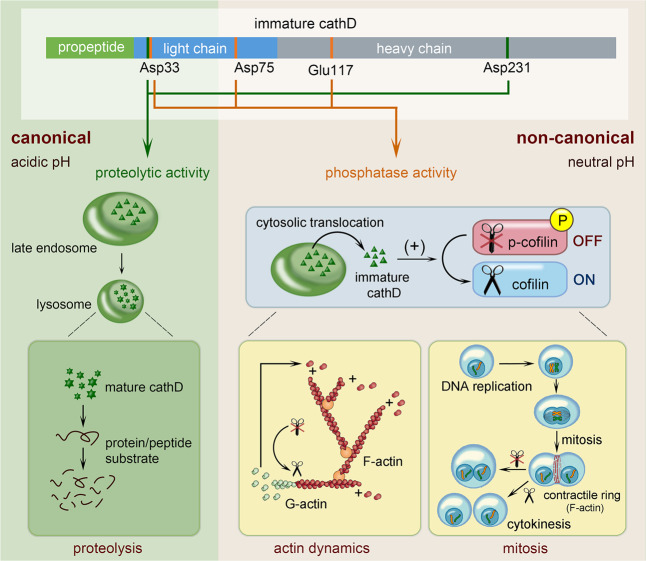
Schematic diagram illustrating the dual role of cathD. In acidic organelles, mature cathD hydrolyzes protein and peptide substrates as a canonical protease (left). In neutral pH environments, immature cathD precursor acts as a noncanonical phosphatase to dephosphorylate p-cofilin and modulate cofilin-mediated processes such as actin remodeling and cell mitosis (right).

Emerging evidence suggests non-proteolytic functions of cathD outside acidic compartments. In the present study, we demonstrate that cathD shows a non-canonical cofilin phosphatase activity in neutral pH and regulates cofilin-mediated actin dynamics, cell mitosis and cytokinesis. Our findings identify cathD as a dual function molecule with both proteolytic and phosphatase activities, which can be switched under different pH conditions and conformational states during maturation (Fig. 7).

Based on our recent findings that cathD balances cofilin activity in cell migration after translocation to cytosol,^17^ we reasoned that cytosolic cathD might non-proteolytically regulate cofilin activity. In addition to the binding interaction between cathD and cofilin, we found that recombinant cathD could directly dephosphorylate cofilin at its Ser3 residue in neutral pH in vitro. In vivo, cathD-deficient flies exhibit cofilin hyperphosphorylation, accompanied by developmental defects in actin-based structures. Similar defects have been previously reported in the absence of cofilin phosphatases,^19,27^ or in the presence of JAS.^31^ Notably, defects in actin-based structures are rescued when reintroducing *cathD* or the *Drosophila* cofilin homolog *tsr* into *cathD*^*1*^ mutants. Together with our results that cathD exhibits ssh-like phosphatase activity in cofilin activation, these observations highlight that cathD functions similarly to ssh in tuning cofilin-mediated actin remodeling.

Dual-role enzymes generally function through similar catalytic mechanisms.^32–34^ Strikingly, proteolytically inactive mutant cathD^D231N^ also dephosphorylate cofilin in neutral pH, suggesting that the phosphatase property of cathD is independent on its proteolytic activity. As potent as the wild-type cathD, proteolytic inactive cathD^D231N^ abolishes cofilin hyperphosphorylation and rescues actin-based malformations induced by cathD depletion. In addition, proteolytically inactive cathD^D231N^ complements ssh action similarly to the wild-type cathD. Therefore, cathD exerts phosphatase and proteolytic activities through different catalytic mechanisms, and these two activities are mutually exclusive.

Enzyme precursors have been thought to be catalytically inactive. Our studies expand this notion by showing that protease precursors, as exemplified by immature cathD, may also possess catalytic activities. The proteolytically inactive immature cathD requires acidic pH and hydrolysis to change conformation and convert to the proteolytically active form.^1,35,36^ In various cell types, the majority of cathD and other cathepsins exist in zymogen forms,^37–39^ while their interaction partners are diversely distributed among different cellular compartments outside the lysosomes.^40^ Our findings uncover that immature cathD and possibly other immature cathepsins may exert broader functions beyond the regulation of cytoskeleton assembly and mitosis. In addition, as cofilin is diversely distributed among different cellular compartments outside the lysosomes, other potential functions of cathD merit further exploration.

In catalytic processes, polar residuals in substrate-binding groove are required for nucleophilic attacks and group transfer reactions.^18,41^ By site-directed mutagenesis, we identified three critical residues (Asp33, Asp75, and Glu117) mediating the cofilin phosphatase activity of cathD. CathD carrying mutation on any of these three residues does not efficiently dephosphorylate cofilin, resulting in elevated cofilin phosphorylation level. Notably, among these residues, Asp33 is also critical for the proteolytic activity of cathD. As the ionization of Asp33 does not require acidic pH environments,^42^ Asp33 likely activates water molecules for the subsequent substrate attack (e.g. removal of a phosphate group) in neutral pH environments. Moreover, our experiments show that cathD^D33G^, cathD^D75G^, and cathD^E117G^ variants exhibit impaired phosphatase activities and induce the generation of giant multinucleated cells with a high rate of aneuploidy. In contrast to wild-type cathD and its proteolytically inactive cathD^D231N^, overexpression of phosphatase-inactive cathD^D33G^, cathD^D75G^, and cathD^E117G^ suppresses A549 cell mitosis and proliferation. Together with previous reports,^9,14^ our study further provides a mechanistic insight into the mitogenicity of cathD, which relies on its cofilin phosphatase activity but not canonical proteolytic activity, in promoting tumor cell proliferation in related cancers.

## Material and methods

### General reagents and antibodies

Antibodies and dyes for immunofluorescence and Western blotting are listed in Supplementary information, Table S1. All other reagents were from Sigma-Aldrich and Roche. Cells were obtained from American Type Culture Collection (ATCC) and Takara Bio Inc. (Takara Bio).

### *Drosophila* strains and genetics

All flies were reared on standard corn meal medium with 50% humidity and 12 h light/12 h dark cycles at 25 °C. *UAS-cathD*^*wt*^ and *UAS-cathD*^*D231N*^ were generated by Prof. Margaret S. Ho. *w*^*1118*^, *cathD*^*1*^ (gift from Dr. Mel Feany), *ssh*^1–63^ mutant flies were kindly gifts from Prof. Tadashi Uemura. *FRT (42B) ubi-GFP* and *hs-flp; FRT (42B)* were obtained from Prof. Zhao-Hui Wang. *UAS-ssh*^*RNAi*^ (107998) was supplied by the Vienna *Drosophila* Resource Center (VDRC). *UAS-ssh*^*wt*^ (BL9113), *UAS-ssh*^*cs*^ (BL9115), *UAS-tsr*^*wt*^ (BL9234), *UAS-tsr*^*S3A*^ (BL9236), *UAS-cathD*^*RNAi*^, and *da-Gal4* (Bloomington *Drosophila* Stock Center) were provided by Bloomington *Drosophila* Stock Center. *UAS-cathD*^*wt*^, *UAS-cathD*^*D231N*^, *UAS-cathD*^*D33G*^, *UAS-cathD*^*D75G*^, *UAS-cathD*^*E117G*^ were generated by microinjection service from the Core Facility of *Drosophila* Resource and Technology, Shanghai Institute of Biochemistry and Cell Biology, Chinese Academy of Sciences.

### Cell culture and transfection

HEK293T, HeLa (ATCC), A549 (ATCC), and Lenti-X 293T (Takara Bio) cell lines were cultured in 10% fetal bovine serum (FBS, Thermo Fisher Scientific) in Dulbecco’s Modified Eagle’s Medium (DMEM, Thermo Fisher Scientific) with high glucose (4.5 g/L). Transient transfections were performed using TurboFect Transfection Reagent (Thermo Fisher Scientific). For Stable transfections, lentivirus was transduced into HEK293 or HeLa cell lines and selected with puromycin (Takara Bio).

### Molecular cloning and plasmid constructions

Full-length human cathD and cathD carrying its proteolytically inactive mutation on Asp231 were cloned from corresponding pcDNA3.1 vectors provided by Prof. Judith Haendeler. To create cathD mutations, Asp and Glu residues in the substrate-binding cleft were altered to Gly by site-directed mutagenesis. Mutagenic primers were designed to target Glu5, Asp12, Asp33, Asp50, Asp75, Glu117, Asp132, and Asp310 residues in Supplementary information, Table S2.

Digested by restriction enzymes *Xho*I and *EcoR*I (Thermo Fisher), PCR fragments were inserted into the pcDNA 3.1/myc-His vector for transient transfection or a pLVX-Puro vector for lentiviral expression. For CRISPR/Cas9-based gene editing, sgRNA-encoding oligonucleotides were designed, synthesized and cloned into LentiCRISPRv2-GFP lentiviral vector to express Cas9, GFP, and sgRNA-targeting cathD.

### Lentivirus production and stable cell line establishment

pLVX-Puro-cathD^wt^, LentiCRISPRv2-cathD sgRNA-GFP, or other cathD mutated constructs were transfected along with the packaging plasmid, psPax and pMD2.G envelop plasmid, into Lenti-X 293T cells at a ratio of 4:3:1. After transfection, Lenti-X 293 T cells were cultured in DMEM with high glucose (4.5 g/L) supplemented with 20% FBS for 36 h. Viral supernatants were then collected by centrifugation at 2000× *g* for 5 min and filtered by 0.45 μm low-protein binding filters (Merck). Harvested viral medium were added with 10 μM polybrene (Sigma-Aldrich) to improve transduction efficiency, diluted with DMEM plus 10% FBS (1:1 in volume) and added to HeLa or HEK293T cells.

After 36–48 h of lentiviral transduction, GFP-positive cells were sorted by a MoFlo Astrios EQ cell sorter (Beckman, Brea, CA) to establish stable cathD-deficient (*cathD*^-/-^) cell line. To further reintroduce cathD^wt^ and other cathD mutants, cathD-contained lentiviral particles were added into *cathD*^-/-^ HeLa cells. Transduction medium was then replaced with fresh culture medium supplemented with puromycin (Takara Bio) to a final concentration of 0.5–3 μg/mL after 48 h of lentiviral transductions to establish stable cathD-mutated cell lines.

### Recombinant protein purification

Purified from the *E. coli*-based expression system, recombinant cathD is prone to aggregate into insoluble inclusion bodies. Therefore, we applied a mammalian expression system to produce recombinant proteins from transient or stably transfected HEK293T cells. Cells were collected and disrupted in 5 mM imdazole, 500 mM NaCl, and 20 mM Tris-HCl (pH 7.9) by sonication. His-tagged cofilin, cathD and its mutated proteins were subsequently purified under native conditions by standard affinity chromatography procedures using a purification resin (Roche Applied Science). Eluted proteins were desalted and concentrated twice by sequential centrifugation using 100-kDa and 50-kDa ultra centrifugal filters (Merck). The molecular weights of purified proteins were checked by silver staining following SDS-PAGE separation for quality control. The protein stability and the secondary structure of mutated cathD proteins were evaluated by a circular dichroism spectroscopy. Purified cathD was mainly in the 48-kDa immature form and stored at −80 °C in 150 mM NaCl, pH 7.4, 0.05% sodium azide and 25% glycerol.

### Phosphatase and protease assays

Purified cathD or its mutations were mixed with cofilin or other candidate proteins in HEPES 50 mM, MgCl_2_ 10 mM, MnCl_2_ 2 mM, 0.2 mM DTT, and 0.1 g/mL BSA at pH 7.5 or pH 3.5, respectively for in vitro phosphatase and protease assays. Mixtures were incubated in 20 μL at 37 °C for 0.5–2 h. To evaluate the enzymatic property of cathD, classical phosphatase inhibitors okadaic acid (1 μM), calyculin A (10 μM), fenvalerate (100 nM), α-naphthyl acid phosphate (10 mM), PhosStop inhibitor cocktail (1:1000), or control vehicles were applied. Reactions were stopped by boiling in SDS-PAGE sample loading buffer (50 mM Tris-HCl, pH 6.8, 2% SDS, 0.1% bromophenol blue, 10% glycerol, 5% β-mercapto-ethanol) for 10 min.

### Pull-down assay

Pull-down experiments were conducted by adding 10 μg purified his-tagged recombinant cathD^wt^ or other cathD mutant proteins into 1 mL whole cell lysate from HEK293 cells (1 × 10^7^ cells). The mixture was incubated with His-Tag purification resin (Sigma) in a binding buffer (20 mM sodium phosphate, 300 mM sodium chloride, 10 mM imidazole, pH 7.4) at 4 °C for 6 h. After washing for three times with a wash buffer (20 mM sodium phosphate, 300 mM sodium chloride, 15 mM imidazole, pH 7.4), the binding partner of cathD and its mutant proteins were resolved with 200 μL elution buffer (20 mM sodium phosphate, 300 mM sodium chloride, 150 mM imidazole, pH 7.4) and examined by Western blotting.

### Cell fractionation and cytosol isolation

The cytosolic fractions were isolated according to previously described protocols.^43^ Briefly, HeLa cells were collected in homogenization buffer (220 mM D-mannitol, 70 mM D-sucrose, 5 mM HEPES-KOH, 1 mM EGTA-KOH, pH 7.4) and homogenized 6 times before centrifuged at 2000× *g* for 10 min. The collected supernatant (S1) was centrifuged at 17,000× *g* for 12 min to remove major organelles (P1). The collected supernatant (S2) was then centrifuged at 100,000× *g* for 60 min to remove endoplasmic reticulum, Golgi and other cytosolic vesicles (P2). The resulting supernatant (S3, cytosolic fractions) was collected and further analyzed with Western blotting.

### Western blot

Protein samples from adult flies were obtained through homogenization of 4 adults per sample using cold RIPA buffer (50 mM Tris, pH 7.4, 150 mM NaCl, 1% Triton X-100, 1% sodium deoxycholate, 0.1% SDS) containing Complete Protease Inhibitor Cocktail and PhosStop inhibitor cocktail (Roche Applied Science).^44^ Heat-shock protein 70 (Hsp70), a stress-inducible chaperone, stabilizes endosome/lysosomes by inhibiting the membrane permeability.^45^ To detect endogenous proteins in cathD and its mutations, stable transfected cell lines were pretreated with 5 μM HSP inhibitor I, a potent inhibitor for hsp70 which could sensitize endosomal/lysosomal membrane and enhance potential cytosolic translocation, for 24 h. Cell lysates were prepared by brief sonication in RIPA buffer on ice, followed by centrifugation at 15,000× rpm for 5 min. The concentration of samples was determined by bicinchoninic acid (BCA) assay and equal amounts of proteins were loaded onto 12% SDS-PAGE gels, followed by transferring for Western blotting. Blots were probed overnight at 4 °C with indicated antibodies in Supplementary information, Table S1.

### Generation of MARCM clones and immunohistochemistry

To generate and stain MARCM clones, the cathD-deleted mutation was recombined onto the FRT (42B) chromosome for MARCM analysis by heat shock twice at 37 °C for 60 min at embryonic day 2. Heat-shocked 3rd instar larval wing discs were dissected in phosphate-buffered saline (PBS) and fixed with 4% paraformaldehyde at room temperature for 20 min. Following permeabilization with 0.3% Triton X-100 for 20 min, tissue was blocked with 4% BSA containing 0.1% Triton X-100 in PBS for 1 h at room temperature and were incubated with primary antibodies (Supplementary information, Table S1) overnight at 4 °C. Alexa Fluor 647-conjugated donkey anti-rabbit secondary antibody was applied (1:1000, Thermo Fisher Scientific).

For F-actin staining, pupal notum from cathD mutants and other genotypes at about 34 h APF were dissected, fixed with 4% paraformaldehyde, and labeled with phalloidin-conjugated with Alexa Fluor 555 or rhodamine (Thermo Fisher Scientific) for 2 h at room temperature.

### Cell synchronization and immunocytochemistry

For synchronization, HeLa and A549 cells were cultured with 2 mM thymidine (Sigma) for 24 h, released for 6 h in fresh complete medium, and treated with 100 ng/mL nocodazole (Sigma) for 6 h. Mitotic cells were collected by shaking off gently, washed with HBSS twice, placed on coverslips in complete medium supplied with 15 μM HSP inhibitor I (Merck), and cultured for 48 h. Following fixation and permeabilization, cells were blocked with 10% BSA and further incubated with Ki67 antibody (1:1000, Abcam) or rhodamine-conjugated phalloidin (1:200, Thermo Fisher Scientific) for 2 h at room temperature. DAPI was applied for nucleus counterstaining. For Ki67 staining, cells were considered as positive when co-stained with DAPI in nucleoli. Images were captured by a confocal microscope (Olympus, FV1200).

### Scanning electron microscopy

Actin-based structures on the adult flies with *cathD*^*1*^ or other mutations were determined by scanning electron microscope (Hitachi, TM-1000). Live flies were anaesthetized with 100% ether and affixed upon stubs. After completely dehydration and air dry, samples were sputter coated with tungsten-platinum by an ion sputter coater (Hitachi, MC1000) and imaged.

### CCK-8 cell proliferation assay

The CCK-8 assay was applied to assess the proliferative activity of A549 cells using a CCK-8 kit (Sigma-Aldrich). Briefly, A549 cells (4 × 10^3^ cells/well) carrying different cathD mutations were seeded in a 96-well plate. After 0, 24, 48, or 72 h, CCK-8 solution (10 μL in 100 μL HBSS) was added and incubated for 2 h at 37 °C. Subsequently, the absorbance of the formazan at 450 nm was measured using a microplate reader (Bio-Rad, iMark) to evaluate A549 cell proliferation.

### Xenograft study

Male athymic nude mice (*nu/nu*, 4 weeks old, obtained from Shanghai Laboratory Animal Center, Chinese Academy of Sciences) were raised under specific pathogen-free conditions and used in tumor xenograft studies. For xenograft transplantation, A549 cells overexpressing cathD^wt^ or mutations on cathD Asp231, Asp33, Asp75G, and Glu117 residues were subcutaneously injected at the left abdomen flank with a density of 1 × 10^7^ cells/mL in 200 μL of Matrigel (Corning, 1:1 volume Matrigel/A549 cells). After injection, animals were individually housed and monitored for 95 days before sacrifice in accordance with the institutional humanitarian regulations.

### Statistical analysis

Statistical analysis was performed using unpaired student *t-*test, Mann–Whitney U test, Kruskal–Wallis test, and one-way ANOVA followed by Tukey’s or Fisher’s LSD post hoc test when appropriate (Supplementary information, Table S3). Data were presented as means ± SEM. *P* < 0.05 was considered to be statistically significant.

## Supplementary information

Fig. S1

Fig. S2

Fig. S3

Fig. S4

Fig. S5

Fig. S6

Table S1

Table S2

Table S3
