# Supplementary material for: The noncanonical role of the protease cathepsin D as a cofilin phosphatase
Source: Cell Res. 2021 Jan 29;31(7):801–13. doi: 10.1038/s41422-020-00454-w (PMC8249557; doi:10.1038/s41422-020-00454-w)
Supplement: Supplementary file 1 — Fig. S1 [file 41422_2020_454_MOESM1_ESM.docx]

**
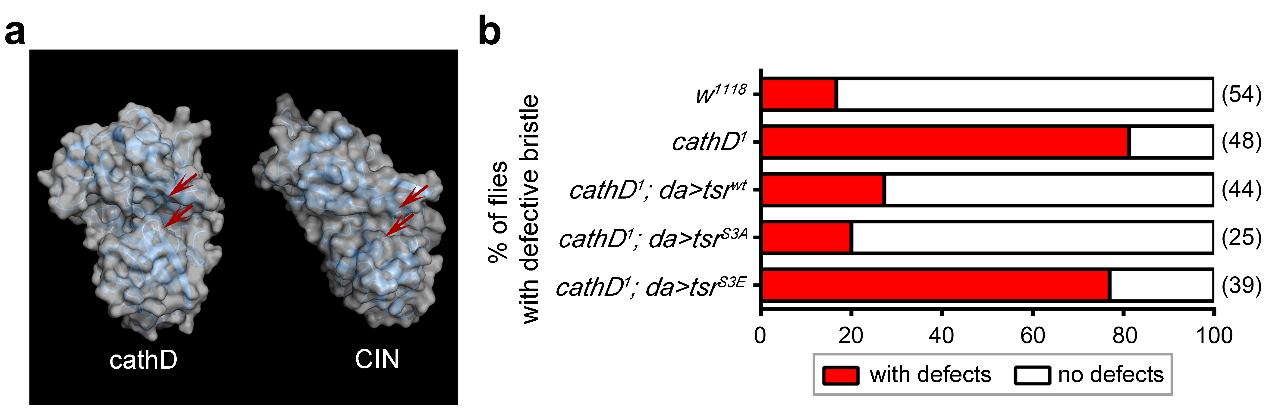
Supplementary information, Fig. S1.** **Loss of cathD increases defective bristles with disordered actin bundles.** **a,** Protein structure of human cathepsin D (cathD; PDB code 1LYB) and chronophin (CIN; PDB code 2cft), showing a similarity in the structures of substrate-binding grooves (arrows indicate paired aspartic residues for nucleophilic attack). **b,** The ratio of individual flies with disorganized actin bundles in bristles in each group. Ubiquitous expression of either tsr^wt^ or tsr^S3A^ in the *cathD^1^* mutant background (*cathD^1^; da>tsr^wt^* or *cathD^1^; DA>tsr^S3A^*) reduces the defective ratio, whereas expression of the constitutively inactive mutant tsr^S3E^ (*cathD^1^; da>tsr^wt^* or *cathD^1^; DA>tsr^S3E^*) exerts minimal effect.
