# Supplementary material for: The noncanonical role of the protease cathepsin D as a cofilin phosphatase
Source: Cell Res. 2021 Jan 29;31(7):801–13. doi: 10.1038/s41422-020-00454-w (PMC8249557; doi:10.1038/s41422-020-00454-w)
Supplement: Supplementary file 2 — Fig. S2 [file 41422_2020_454_MOESM2_ESM.docx]

**
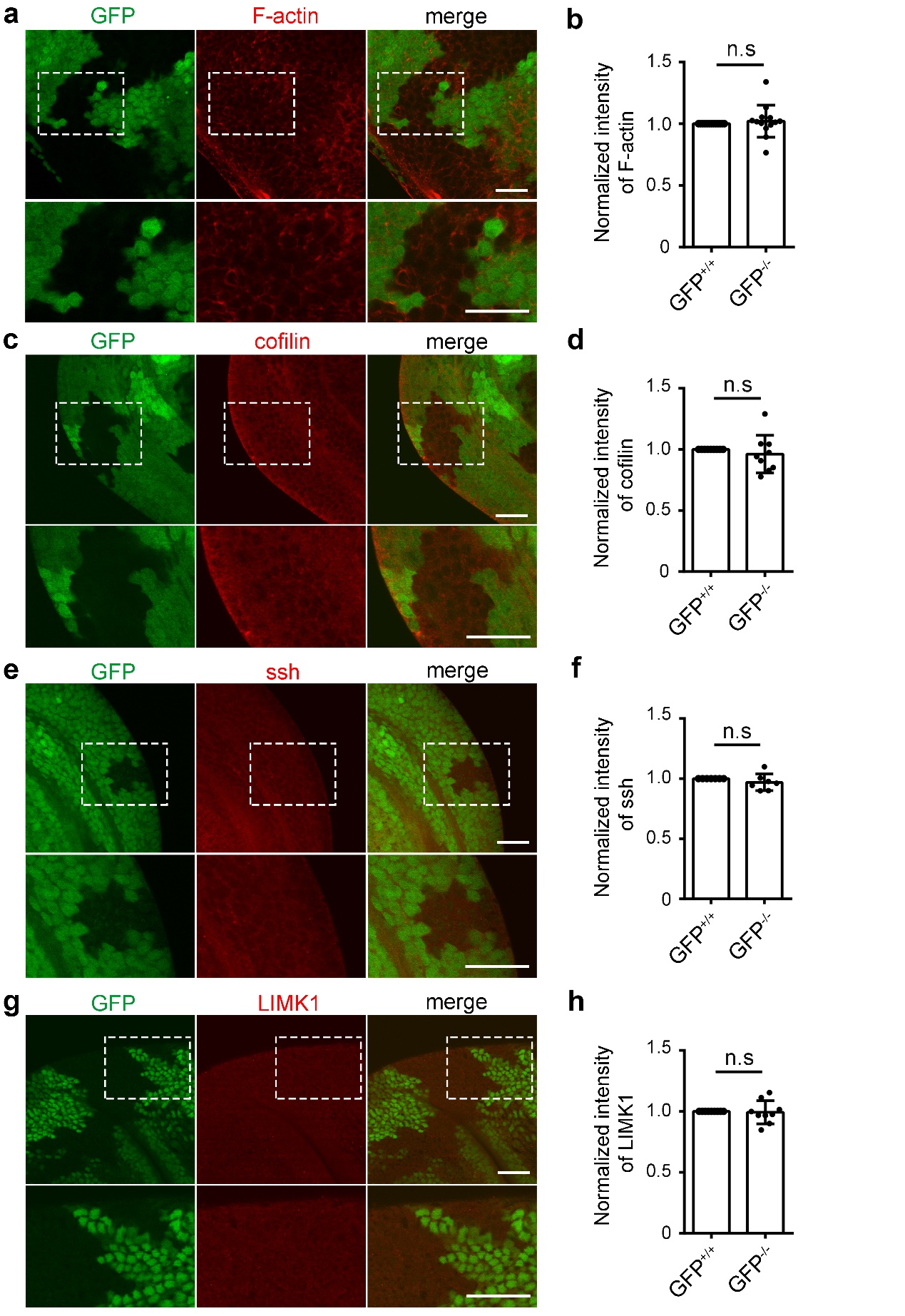
Supplementary information, Fig. S2.** **Loss of cathD does not change F-actin, cofilin, ssh, or LIMK1 levels in larval wing disc development. a-h,** *cathD^1^* mutant clones (GFP-negative) were generated using FRT recombination in the 3^rd^ instar larval wing discs. Representative images (a, c, e, g) and corresponding quantification (b, d, f, h) showing that fluorescence intensities of F-actin (a, b), cofilin (c, d), ssh (e, f), and LIMK1/2 (g, h) are not significantly changed in *cathD^1^*mutant clones (GFP negative), compared with adjacent twinspot wild-type clones (GFP-positive). Scale bars, 20 μm.
