# Supplementary material for: The noncanonical role of the protease cathepsin D as a cofilin phosphatase
Source: Cell Res. 2021 Jan 29;31(7):801–13. doi: 10.1038/s41422-020-00454-w (PMC8249557; doi:10.1038/s41422-020-00454-w)
Supplement: Supplementary file 3 — Fig. S3 [file 41422_2020_454_MOESM3_ESM.docx]

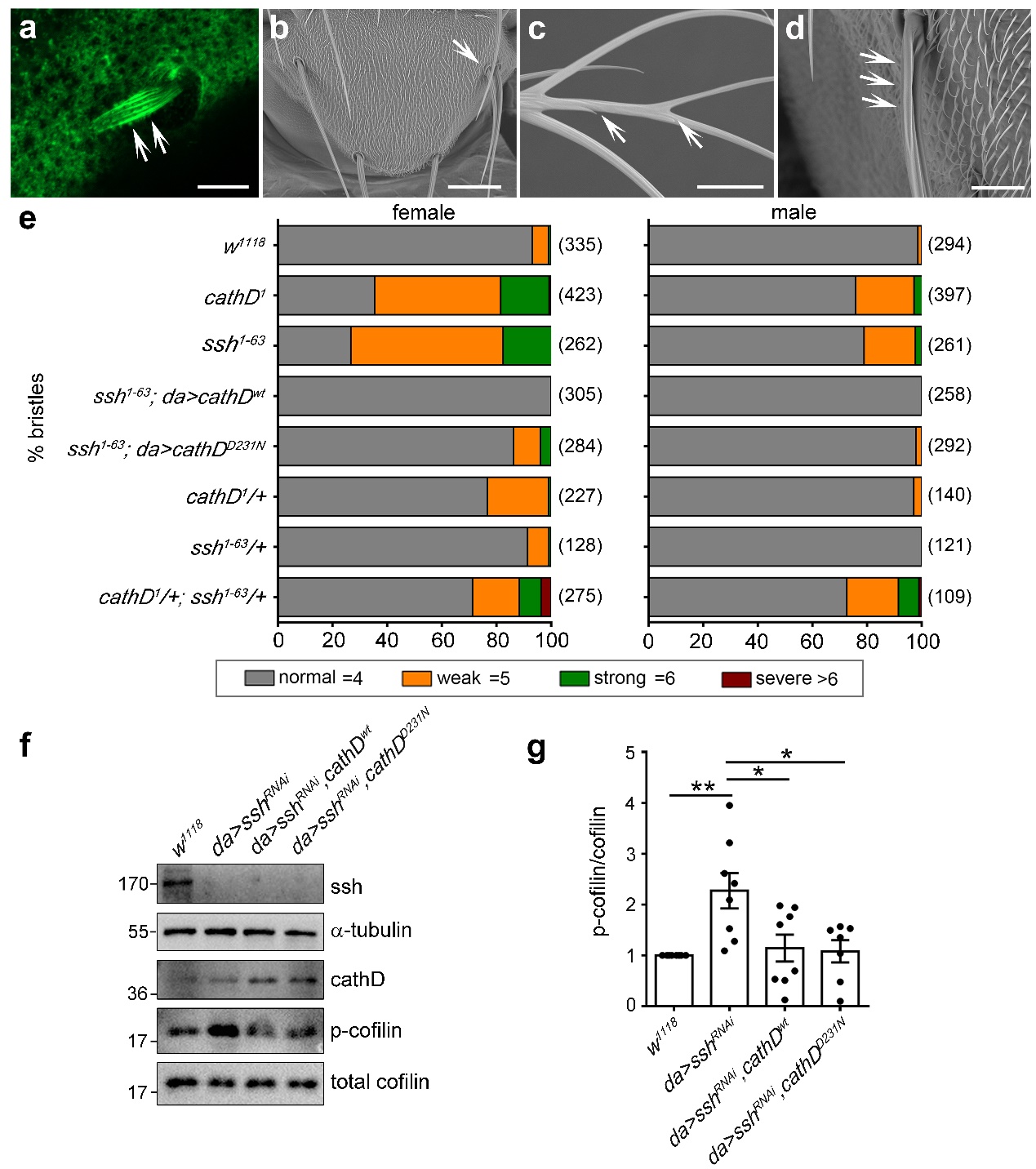
**Supplementary information, Fig. S3.** **CathD exhibits slingshot-like phosphatase activity. a-d,** Representative confocal and SEM images show actin-based malformations in *ssh^1-63^* mutant flies. Defects in *ssh*-deficient animals include: **a,** phalloidin-labeled protrusion-like structures at bristle tips at 34 hr APF (arrows indicate microvillar-like short protrusions); **b,** extra bristles on the scutellum (the arrow indicates an ectopic bristle); **c,** increased lateral branches on antenna (arrows indicate split laterals); and **d,** scutellar bristle malformations (arrows indicate disorganized bridges). Scale bars, 10 μm for a and c, 40 μm for b, and 20 μm for d. **e,** Analysis and severity-based classification of scutellar bristles in adult flies, showing that expression of wild-type cathD (*ssh^1-63^; da>cathD^wt^*) or its proteolytically inactive mutant (*ssh^1-63^; da>cathD^D231N^*) in the presence of *ssh^1-63^* mutants dramatically decreases the number of ectopic bristles in both genders, whereas transheterozygotes of *cathD^1^* and *ssh^1-63^* (*cathD^1^/+; ssh^1-63^/+*) exhibits stronger phenotypes than either heterozygote (*cathD^1^/+* and *ssh^1-63^/+*). Data are collected from 3 separate experiments. **f, g,** Representative immunoblot images (f) and quantification (g) of total protein extracted from *ssh* knockdown (*da>ssh^RNAi^*) adults flies, showing elevated p-cofilin levels in *da>ssh^RNAi^* flies. Expression of wild-type cathD or its proteolytically inactive mutant cathD^D231N^ restores cofilin hyperphosphorylation in *da>ssh^RNAi^* mutants. Data shown are representative of 8 independent experiments. Data are mean ± S.E.M.. One-way ANOVA with Tukey's *post hoc* test *P < 0.05, **P < 0.01.
