# Supplementary material for: The noncanonical role of the protease cathepsin D as a cofilin phosphatase
Source: Cell Res. 2021 Jan 29;31(7):801–13. doi: 10.1038/s41422-020-00454-w (PMC8249557; doi:10.1038/s41422-020-00454-w)
Supplement: Supplementary file 4 — Fig. S4 [file 41422_2020_454_MOESM4_ESM.docx]

**
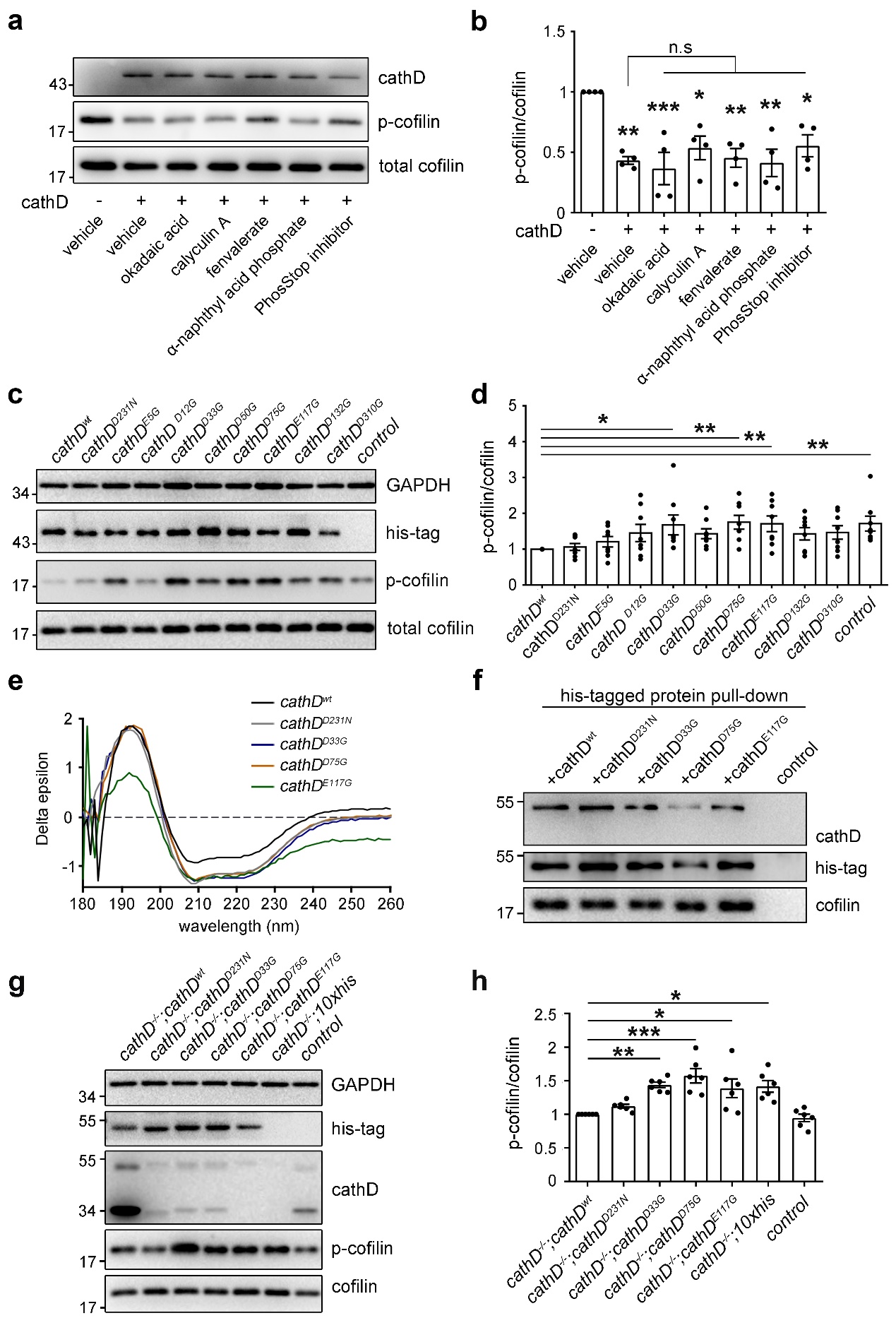
Supplementary information, Fig. S4.** **Asp33, Asp75 and Glu117 are essential for the phosphatase activity of cathD.** **a, b,** Representative immunoblot images (a) and quantification (b) from *in vitro* phosphatase assays on the cofilin phosphatase activity of cathD, showing that recombinant cathD protein dephosphorylates purified cofilin in the presence of classic phosphatase inhibitors without exerting inhibitory effects among groups. **c, d,** Single mutations at Asp/Glu amino acid positions (5, 12, 33, 50, 75, 117, 132, or 310) of human cathD were created by PCR-directed mutagenesis. Each histidine (his)- tagged mutation was transfected and expressed in HEK293T cells to generate stable cell lines. Representative Western blots of total cell lysates from cathD-mutated and control cell lines (c). Note that the expression of cathD^D33G^, cathD^D75G^ and cathD^E117G^ mutants increases p-cofilin levels. Quantifications of p-cofilin/cofilin ratio (normalized to cathD^wt^) are shown (d). Note a significant increase in ratio upon expression of cathD^D33G^, cathD^D75G^ or cathD^E117G^ mutants. **e,** Circular dichroism spectroscopy analysis of cathD^wt^, cathD^D231N^, cathD^D33G^, cathD^D75G^, or cathD^E117G^ proteins, showing no significant changes among different mutants. **f,** Direct interaction between cathD and cofilin. Representative immunoblot images show that his-tagged cathD^wt^ and its mutants directly bind cofilin from HEK293 cells, showing no differences in cofilin binding affinity among cathDwt and its mutants on Asp 33, Asp75, Glu117, nor Asp231. **g, h,** cathD^-/-^ HEK293T cells were reintroduced with indicated mutants and treated with 5 μM Hsp inhibitor I for 24 hr before harvest. Representative immunoblot images (g) and quantification of p-cofilin/cofilin ratio (normalized to cathD^wt^, h) show that reintroducing cathD^D33G^, cathD^D75G^ or cathD^E117G^, but not cathD^wt^ or cathD^D231N^, increases phosphorylated cofilin levels. Data are shown as mean ± S.E.M.. One-way ANOVA with Tukey's post hoc test with significance *P < 0.05, **P < 0.01 and *** p<0.001.
