# Supplementary material for: The noncanonical role of the protease cathepsin D as a cofilin phosphatase
Source: Cell Res. 2021 Jan 29;31(7):801–13. doi: 10.1038/s41422-020-00454-w (PMC8249557; doi:10.1038/s41422-020-00454-w)
Supplement: Supplementary file 5 — Fig. S5 [file 41422_2020_454_MOESM5_ESM.docx]

**
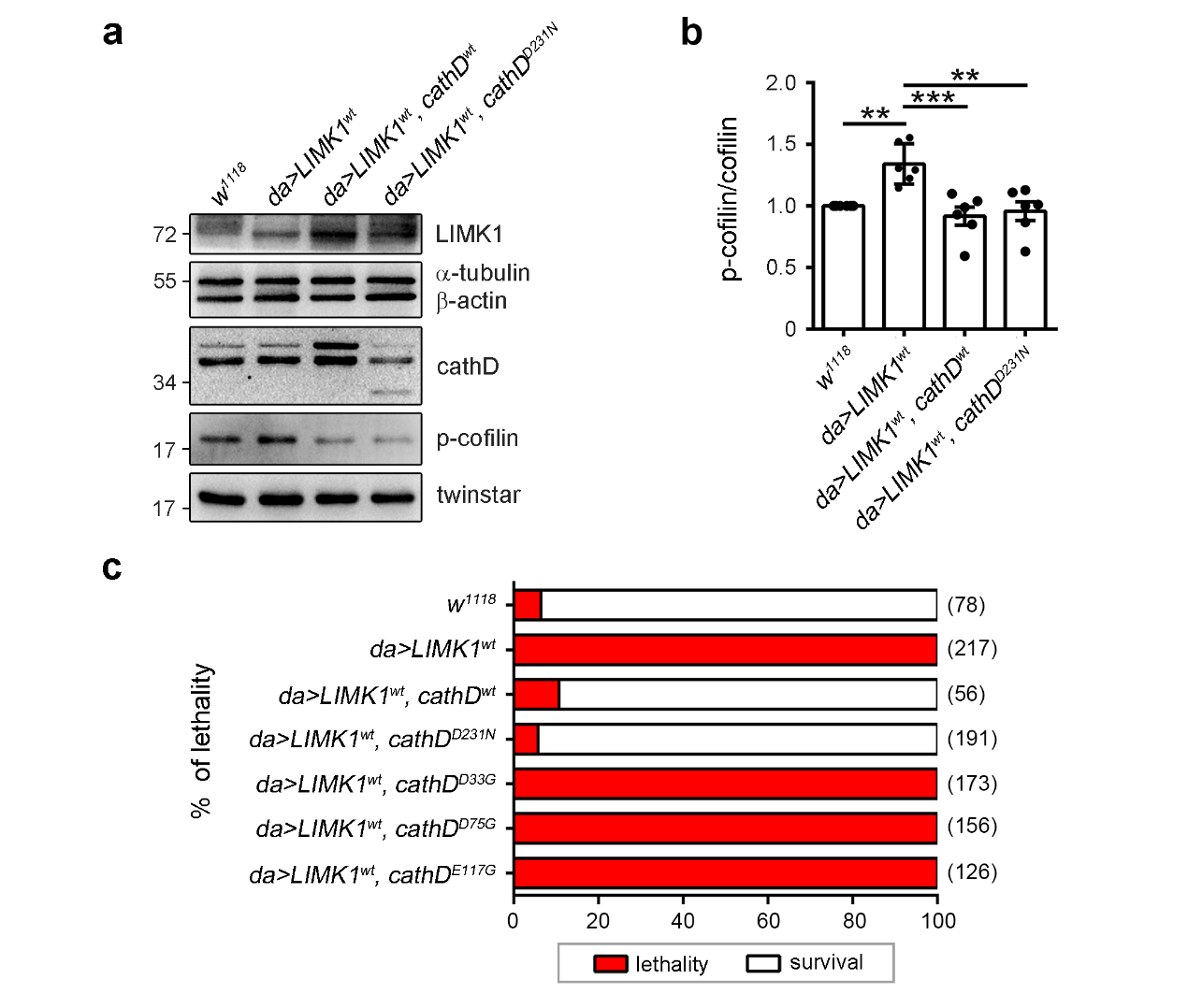
Supplementary information, Fig. S5.** CathD non-proteolytically restores cofilin hyperphosphorylation and lethality in LIMK1-overexpression flies. **a, b,** Representative immunoblot images (a) and quantifications (b) of total protein extracted from 3^rd^ instar larvae. (a) Overexpression of LIMK1 (*da>LIMK1^wt^*) increases p-cofilin levels, which are restored upon expressing either wild-type (*da>LIMK1^wt^, cathD^wt^*) or proteolytically inactive cathD (*da>LIMK1^wt^, cathD^D231N^*). (b) Quantification of p-cofilin/total cofilin levels (normalized to *w^1118^* controls). Data shown are representative of 6 independent experiments. **c,** Ubiquitous overexpression of LIMK1 (*da>LIMK1^wt^*) causes lethality in flies before eclosion. Quantification of lethality rate in adult flies shows that co-expressing either wild-type (*da>LIMK1^wt^, cathD^wt^*) or proteolytically inactive cathD (*da>LIMK1^wt^, cathD^D231N^*) decreases the lethality caused by LIMK1 overexpression (*da>LIMK1^wt^*), whereas expression of phosphatase inactive cathD^D33G^ (*da>LIMK1^wt^, cathD^D33G^*), cathD^D75G^ (*da>LIMK1^wt^, cathD^D75G^*), or cathD^E117G^ (*LIMK1^wt^, cathD^E117G^*) shows no effects. Data are mean ± S.E.M.. One-way ANOVA with Newman-Keuls *post hoc* test. **P < 0.01, ***P < 0.001.
