# Supplementary material for: The noncanonical role of the protease cathepsin D as a cofilin phosphatase
Source: Cell Res. 2021 Jan 29;31(7):801–13. doi: 10.1038/s41422-020-00454-w (PMC8249557; doi:10.1038/s41422-020-00454-w)
Supplement: Supplementary file 6 — Fig. S6 [file 41422_2020_454_MOESM6_ESM.docx]

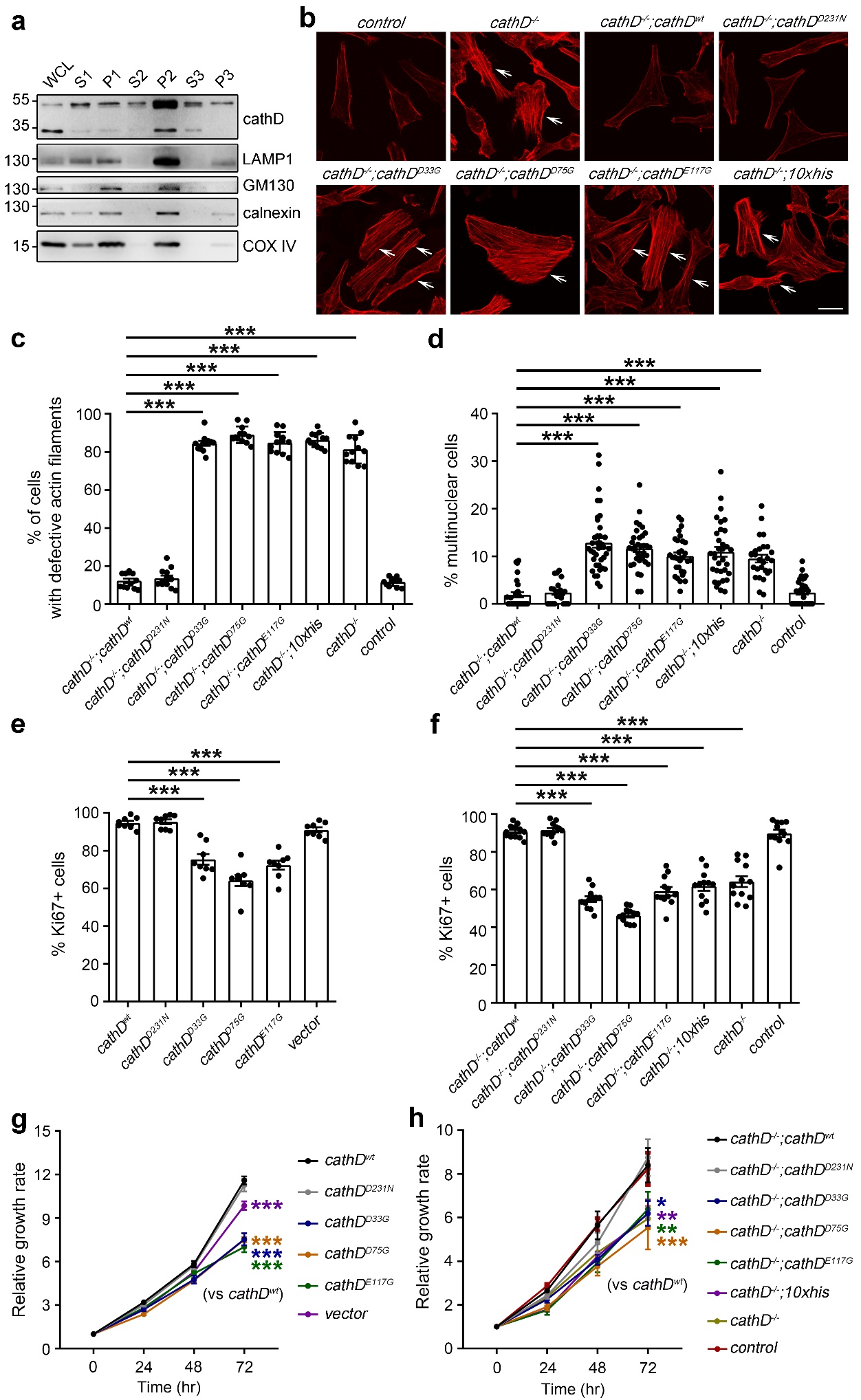
**Supplementary information, Fig. S6.** **Asp33, Asp75, and Glu117 of cathD are essential for cytokinesis and cell proliferation.** **a,** HeLa cells were fractionated into six distinct fractions after differential centrifugation, yielding whole cell lysis (WCL), debris and unbroken cells (P1), supernatant containing cytosol and organelles (S1), major vesicles (P2), supernatant containing cytosol and small organelles (S2), small organelles and membrane (P3), and cytoplasm (S3). S, supernatant; P, pellet. Fractions were analyzed by immunoblotting using indicated antibodies. A representative blot shows that immature cathD is its main form in the cytosolic fraction (S3). **b, c,** Representative F-actin staining images (b) and quantifications (c) show defective actin patterns in cathD-mutated HeLa cells. Arrows indicate strong signals of puncta or fibers at cell periphery or within cell bodies. Note that cathD mutation on Asp33, Asp75 or Glu117 increases the number of cells with defective actin filaments (c). **d,** Quantifications of multinuclear cells. CathD^-/-^ HeLa were reintroduced with indicated cathD mutants. Compared with cathD^wt^ or proteolytically inactive cathD^D231N^, reintroduction of cathD^D33G^, cathD^D75G^, cathD^E117G^ failed to reduce the number of giant multinuclear cells. **e, f,** Quantifications of Ki67-positive HeLa cells with indicated cathD mutants. **g, h,** CCK-8 assay of HeLa cells at indicated time points. Relative proliferation ratios were normalized to the values at 0 hr. Data are mean ± S.E.M.. One-way ANOVA followed with Tukey's test, or two-way ANOVA followed with Dunnett 's test, * p<0.05, ** p<0.01 and *** p<0.001.
