# Supplementary material for: The noncanonical role of the protease cathepsin D as a cofilin phosphatase
Source: Cell Res. 2021 Jan 29;31(7):801–13. doi: 10.1038/s41422-020-00454-w (PMC8249557; doi:10.1038/s41422-020-00454-w)
Supplement: Supplementary file 7 — Table S1 [file 41422_2020_454_MOESM7_ESM.docx]

**Supplementary information, Table S1. Summary of antibodies and dyes used in experiments**

| **Antibodies/Dyes** | **Source** | **Catalogue #** | **Host** | **Dilution** |
| --- | --- | --- | --- | --- |
| Anti-p-cofilin (Phospho-Ser3) | SAB Biotech | 11139-2 | Rabbit | IF: 1/200 |
|  | Santa Cruz Biotechnology | sc-12912-12 | Rabbit | WB: 1/1000 |
| Anti-cofilin | Cell Signaling Technology | 5715 | Rabbit | WB: 1/1000 |
| Anti-β-Actin | Sigma-Aldrich | A1978 | Mouse | WB: 1/1000 |
| Anti-α-Tubulin | Sigma-Aldrich | T5168 | Mouse | WB: 1/3000 |
| Anti-cathepsin D | Santa Cruz Biotechnology | sc-6487 | Goat | WB: 1/1000 |
|  | Abcam | ab75852 | Rabbit | WB: 1/500 |
| Anti-twinstar | SAIER | SRP00895 | Rabbit | WB: 1/3000 |
| Anti-6X His-tag | Abcam | ab9108 | Rabbit | WB: 1/1000 |
| GAPDH | Sigma-Aldrich | G9252 | Mouse | WB: 1/10000 |
| Anti-14-3-3 ζ | EMD Millipore | AB9746 | Rabbit | WB: 1/3000 |
| Anti-p-LIMK1/2 (phospho-T508) | Bioworld Technology | BS4115 | Rabbit | WB: 1/1000 |
| Anti-LIMK1 | Santa Cruz Biotechnology | sc-8387 | Goat | WB: 1/500 |
| Anti-Slingshot homolog 1 | Abcam | ab46202 | Rabbit | WB: 1/1000 |
|  | Abcam | ab76943 | Rabbit | WB: 1/1000 |
| Anti-Ki67 | Abcam | Ab16667 | Rabbit | IF: 1/1000 |
| Anti-calnexin | Cell Signaling Technology | 2679 | Rabbit | WB: 1/1000 |
| Anti-COX IV | Cell Signaling Technology | 4850 | Rabbit | WB: 1/1000 |
| Cy3-AffiniPure donkey anti-rabbit IgG (H+L) | Jackson ImmunoResearch | 711-165-152 | Donkey | IF: 1/1000 |
| Peroxidase AffiniPure Goat Anti-Mouse IgG (H+L) | Jackson ImmunoResearch | 115-035-146 | Goat | WB: 1/10000 |
| Peroxidase AffiniPure Goat Anti-Rabbit IgG (H+L) | Jackson ImmunoResearch | 111-035-144 | Goat | WB: 1/10000 |
| Peroxidase-AffiniPure Donkey Anti-Goat IgG (H+L) | Jackson ImmunoResearch | 705-035-003 | Donkey | WB: 1/10000 |
| Rhodamine Phalloidin | Thermo Fisher Scientific | R415 | - | IF: 1/200 |
| Alexa Fluor 555 Phalloidin | Thermo Fisher Scientific | A34055 | - | IF: 1/200 |
| Donkey-anti-rabbit IgG (H+L), Alexa 647 | Thermo Fisher Scientific | A31573 | Donkey | IF: 1/1000 |
| 4',6-Diamidino-2-Phenylindole, Dihydrochloride (DAPI) | Beyotime Biotechnology | C1006 | - | None |
