# Supplementary material for: The noncanonical role of the protease cathepsin D as a cofilin phosphatase
Source: Cell Res. 2021 Jan 29;31(7):801–13. doi: 10.1038/s41422-020-00454-w (PMC8249557; doi:10.1038/s41422-020-00454-w)
Supplement: Supplementary file 8 — Table S2 [file 41422_2020_454_MOESM8_ESM.docx]

**Supplementary information, Table S2.** **Primers for site directed mutagenesis**

| **Targets** | **Mutations** | **Primers** | **Sequences** |
| --- | --- | --- | --- |
| Glu5 | cathD^E5G^ | sense | 5’ ATTCCCGGGGTGCTCAAGAACTACAT 3’ |
|  |  | antisense | 5’ ATGTAGTTCTTGAGCACCCCGGGAAT 3’ |
| Asp12 | cathD^D12G^ | sense | 5’ TCAAGAACTACATGGGGGCCCAGTACTA 3’ |
|  |  | antisense | 5’ TAGTACTGGGCCCCCATGTAGTTCTTGA 3’ |
| Asp33 | cathD^D33G^ | sense | 5’ ACAGTCGTCTTCGGGACGGGC 3’ |
|  |  | antisense | 5’ GCCCGTCCCGAAGACGACTGT 3’ |
| Asp50 | cathD^D50G^ | sense | 5’ CTGCAAACTGCTGGGGATCGC 3’ |
|  |  | antisense | 5’ GCGATCCCCAGCAGTTTGCAG 3’ |
| Glu117 | cathD^E117G^ | sense | 5’ TCTTTGGGGGGGCCACCAAG 3’ |
|  |  | antisense | 5’ CTTGGTGGCCCCCCCAAAGA 3’ |
| Asp132 | cathD^D132G^ | sense | 5’ AGCCAAGTTCGGGGGCATCCT 3’ |
|  |  | antisense | 5’ AGGATGCCCCCGAACTTGGCT 3’ |
| Asp310 | cathD^D310G^ | sense | 5’ ATGGGCATGGGGATCCCGCC 3’ |
|  |  | antisense | 5’ GGCGGGATCCCCATGCCCAT 3’ |
