# Supplementary material for: The noncanonical role of the protease cathepsin D as a cofilin phosphatase
Source: Cell Res. 2021 Jan 29;31(7):801–13. doi: 10.1038/s41422-020-00454-w (PMC8249557; doi:10.1038/s41422-020-00454-w)
Supplement: Supplementary file 9 — Table S3 [file 41422_2020_454_MOESM9_ESM.docx]

**Supplementary information, Table S3.** **Summary of statistical methods and results**

| Figure. | Statistical method | Statistical results | | P value |
| --- | --- | --- | --- | --- |
| 2f | unpaired student t test | t_16_=5.516 | | <0.001*** |
| 3d | One-way ANOVA | F_(3, 31)_=29.56 | | <0.001*** |
|  | Tukey's *post hoc* test | *w^1118^* vs. *cathD^1^* | | <0.05* |
|  |  | *w^1118^* vs. ssh*^1-63^* | | <0.05* |
|  |  | *w^1118^* vs. *cathD^1^*; ssh*^1-63^* | | <0.001*** |
|  |  | *cathD^1^* vs. *cathD^1^*; ssh*^1-63^* | | <0.001*** |
|  |  | ssh*^1-63^* vs. *cathD^1^*; ssh*^1-63^* | | <0.001*** |
| 3f | One-way ANOVA | F_(3, 31)_=9.507 | | =0.0001**** |
|  | Tukey's *post hoc* test | *w^1118^* vs. *ssh^1-63^* | | <0.001*** |
|  |  | *ssh^1-63^* vs. *ssh^1-63^; da>cathD^wt^* | | <0.01** |
|  |  | *ssh^1-63^* vs. *ssh^1-63^; da>cathD^D231N^* | | <0.01** |
| 3h | One-way ANOVA | F_(3, 30)_= 8.585 | | =0.0003*** |
|  | Tukey's *post hoc* test | w*^1118^* vs. da>ssh^RNAi^ | | <0.05* |
|  |  | da>cathD^RNAi^ vs. da>cathD^RNAi^, ssh^wt^ | | <0.001*** |
| 4b | one-way ANOVA | F _(2, 9)_ = 22.08 | | <0.001*** |
|  | Tukey's *post hoc* test | control vs.cathD^wt^ | | <0.001*** |
|  |  | control vs. cathD^D231N^ | | <0.001*** |
| 4d | One-way ANOVA | F_(3, 28)_=10.88 | | <0.0001**** |
|  | Tukey's *post hoc* test | w^1118^ vs. cathD^1^ | | <0.01** |
|  |  | cathD^1^ vs. cathD^1^;da>cathD^wt^ | | <0.001*** |
|  |  | cathD^1^ vs. cathD^1^;da>cathD^D231N^ | | <0.001*** |
| 4f | one-way ANOVA | F _(2, 9)_ = 9.126 | | = 0.0068** |
|  | Tukey's *post hoc* test | control vs. immature cathD | | <0.01** |
|  |  | mature cathD vs. immature cathD | | <0.05* |
| 4h | one-way ANOVA | F _(5, 30)_ = 14.23 | | <0.001*** |
|  | Tukey's *post hoc* test | cathD^wt^ vs. cathD^D33G^ | | <0.01** |
|  |  | cathD^wt^ vs. cathD^D75G^ | | <0.001*** |
|  |  | cathD^wt^ vs. cathD^E117G^ | | <0.001*** |
|  |  | cathD^wt^ vs. control | | <0.001*** |
| 4i | one-way ANOVA | F _(5, 36)_ = 14.31 | | <0.001*** |
|  | Tukey's *post hoc* test | cathD^wt^ vs. cathD^D231N^ | | <0.01** |
|  |  | cathD^wt^ vs. cathD^D33G^ | | <0.05* |
|  |  | cathD^wt^ vs. control | | <0.001*** |
| 5b | one-way ANOVA | F_(5, 42)_= 9.274 | | <0.0001*** |
|  | Tukey's *post hoc* test | cathD^1^;da>cathD^wt^ vs.cathD^1^ | | <0.001*** |
|  |  | cathD^1^;da>cathD^wt^ vs. cathD^1^;da>cathD^D33G^ | | <0.05* |
|  |  | cathD^1^;da>cathD^wt^ vs. cathD^1^;da>cathD^D75G^ | | <0.01** |
| 6b | Mann-Whitney U test | U=11 | | <0.001*** |
| 6c | Kruskal-Wallis test | H=96.39 | | <0.0001*** |
|  | Dunn's *post hoc* test | cathD^wt^ vs. cathD^D33G^ | | <0.001*** |
|  |  | cathD^wt^ vs. cathD^D75G^ | | <0.001*** |
|  |  | cathD^wt^ vs. cathD^E117G^ | | <0.001*** |
| 6d | one-way ANOVA | F _(5, 31)_ = 27.15 | | <0.0001*** |
|  | Tukey's *post hoc* test | cathD^wt^ vs. cathD^D33G^ | | <0.001*** |
|  |  | cathD^wt^ vs. cathD^D75G^ | | <0.001*** |
|  |  | cathD^wt^ vs. cathD^E117G^ | | <0.01** |
| 6e | two-way ANOVA | Condition effect: F_(12, 120)_ = 11.83 | | <0.0001*** |
|  |  | Time effect: F_(3, 120)_ = 294.3 | | <0.0001*** |
|  |  | Interaction: F_(12, 120)_ = 11.83 | | <0.0001*** |
|  | Tukey's *post hoc* test | 24 hr | cathD^wt^ vs. cathD^D33G^ | <0.001*** |
|  |  |  | cathD^wt^ vs. cathD^D75G^ | <0.01** |
|  |  |  | cathD^wt^ vs. cathD^E117G^ | <0.001*** |
|  |  | 48 hr | cathD^wt^ vs. cathD^D33G^ | <0.001*** |
|  |  |  | cathD^wt^ vs. cathD^D75G^ | <0.001*** |
|  |  |  | cathD^wt^ vs. cathD^E117G^ | <0.001*** |
|  |  | 72 hr | cathD^wt^ vs. cathD^D33G^ | <0.001*** |
|  |  |  | cathD^wt^ vs. cathD^D75G^ | <0.01** |
|  |  |  | cathD^wt^ vs. cathD^E117G^ | <0.01** |
| 6g | two-way ANOVA | Condition effect: F_(17, 594)_ = 16.18 | | <0.0001*** |
|  |  | Time effect: F_(5, 594)_ = 100.7 | | <0.0001*** |
|  |  | Interaction: F_(85, 594)_ = 9.481 | | <0.0001*** |
|  | Tukey's *post hoc* test | cathD^wt^ vs. cathD^D33G^ | | <0.001*** |
|  |  | cathD^wt^ vs. cathD^D75G^ | | <0.001*** |
|  |  | cathD^wt^ vs. cathD^E117G^ | | <0.001*** |
|  |  | cathD^wt^ vs. control | | <0.001*** |
| S3g | One-way ANOVA | F_(3, 27 )_= 6.066 | | =0.0027** |
|  | Tukey's *post hoc* test | *w^1118^* vs. *da>ssh^RNAi^* | | <0.01** |
|  |  | da>ssh^RNAi^ vs. da>ssh^RNAi^, cathD^wt^ | | <0.05* |
|  |  | da>ssh^RNAi^ vs. da>ssh^RNAi^, cathD^D231N^ | | <0.05* |
| S4b | One-way ANOVA | F _(6, 21)_ = 5.807 | | P=0.0011 |
|  | Tukey's *post hoc* test | vehicle vs. vehicle + cathD | | <0.01** |
|  |  | vehicle vs. okadaic acid + cathD | | <0.001*** |
|  |  | vehicle vs. calyculin A + cathD | | <0.05* |
|  |  | vehicle vs. fenvalerate + cathD | | <0.01** |
|  |  | vehicle vs. α-naphthyl acid phosphate + cathD | | <0.01** |
|  |  | PhosStop inhibitor | | <0.05* |
| S4d | one-way ANOVA | F _(10, 77)_ = 2.010 | | =0.0434* |
|  | Fisher’s LSD *hoc* test | cathD^wt^ vs. cathD^D33G^ | | <0.05* |
|  |  | cathD^wt^ vs. cathD^D75G^ | | <0.01** |
|  |  | cathD^wt^ vs. cathD^E117G^ | | <0.01** |
|  |  | cathD^wt^ vs. control | | <0.01** |
| S4h | One-way ANOVA | F _(6, 35)_ = 9.486 | | <0.001*** |
|  | Tukey's *post hoc* test | cathD^-/-^; cathD^wt^ vs. cathD^-/-^; cathD^D33G^ | | <0.01** |
|  |  | cathD^-/-^; cathD^wt^ vs. cathD^-/-^; cathD^D75G^ | | <0.001*** |
|  |  | cathD^-/-^; cathD^wt^ vs. cathD^-/-^; cathD^E117G^ | | <0.05* |
|  |  | cathD^-/-^; cathD^wt^ vs. cathD^-/-^; 10xhis | | <0.05* |
| S5b | One-way ANOVA | F _(3, 20)_= 9.682 | | <0.0001**** |
|  | Tukey's *post hoc* test | *w^1118^* vs. *da>LIMK1^wt^* | | <0.01** |
|  |  | *da>LIMK1^wt^* vs. *da>LIMK1^wt^, cathD^wt^* | | <0.001*** |
|  |  | *da>LIMK1^wt^* vs. *da>LIMK1^wt^, cathD^D231N^* | | <0.01** |
| S6c | One-way ANOVA | F _(7, 88)_ = 735.5 | | <0.001*** |
|  | Tukey's *post hoc* test | cathD^-/-^; cathD^wt^ vs. cathD^-/-^; cathD^D33G^ | | <0.001*** |
|  |  | cathD^-/-^; cathD^wt^ vs. cathD^-/-^; cathD^D75G^ | | <0.001*** |
|  |  | cathD^-/-^; cathD^wt^ vs. cathD^-/-^; cathD^E117G^ | | <0.001*** |
|  |  | cathD^-/-^; cathD^wt^ vs. cathD^-/-^; 10xhis | | <0.001*** |
|  |  | cathD^-/-^; cathD^wt^ vs. cathD^-/-^ | | <0.001*** |
| S6d | One-way ANOVA | F _(7, 246)_ = 19.24 | | <0.0001**** |
|  | Tukey's *post hoc* test | cathD^-/-^; cathD^wt^ vs. cathD^-/-^; cathD^D33G^ | | <0.001*** |
|  |  | cathD^-/-^; cathD^wt^ vs. cathD^-/-^; cathD^D75G^ | | <0.001*** |
|  |  | cathD^-/-^; cathD^wt^ vs. cathD^-/-^; cathD^E117G^ | | <0.001*** |
|  |  | cathD^-/-^; cathD^wt^ vs. cathD^-/-^; 10xhis | | <0.001*** |
|  |  | cathD^-/-^; cathD^wt^ vs. cathD^-/-^ | | <0.001*** |
| S6e | One-way ANOVA | F _(6, 35)_ = 36.89 | | <0.0001**** |
|  | Tukey's *post hoc* test | cathD^wt^ vs. cathD^D33G^ | | <0.001*** |
|  |  | cathD^wt^ vs. cathD^D75G^ | | <0.001*** |
|  |  | cathD^wt^ vs. cathD^E117G^ | | <0.001*** |
| S6f | One-way ANOVA | F _(7, 88)_ = 91.13 | | <0.0001**** |
|  | Tukey's *post hoc* test | cathD^-/-^; cathD^wt^ vs. cathD^-/-^; cathD^D33G^ | | <0.001*** |
|  |  | cathD^-/-^; cathD^wt^ vs. cathD^-/-^; cathD^D75G^ | | <0.001*** |
|  |  | cathD^-/-^; cathD^wt^ vs. cathD^-/-^; cathD^E117G^ | | <0.001*** |
|  |  | cathD^-/-^; cathD^wt^ vs. cathD^-/-^; 10xhis | | <0.001*** |
|  |  | cathD^-/-^; cathD^wt^ vs. cathD^-/-^ | | <0.001*** |
| S6g | two-way ANOVA | Condition effect: F _(5, 144)_ = 85.27 | | <0.0001*** |
|  |  | Time effect: F_(3, 144)_ = 390 | | <0.0001*** |
|  |  | Interaction: F _(15, 144)_ = 10.66 | | <0.0001*** |
|  | Dunnett's *post hoc* test | cathD^wt^ vs. cathD^D33G^ | | <0.001*** |
|  |  | cathD^wt^ vs. cathD^D75G^ | | <0.001*** |
|  |  | cathD^wt^ vs. cathD^E117G^ | | <0.001*** |
|  |  | cathD^wt^ vs. vector | | <0.001*** |
| S6h | two-way ANOVA | Condition effect: F _(3, 256)_ = 7.145 | | <0.0001*** |
|  |  | Time effect: F_(3, 256)_ = 297.1 | | <0.0001*** |
|  |  | Interaction: F _(21, 256)_ = 1.842 | | P=0.0154 |
|  | Dunnett's *post hoc* test | cathD^-/-^; cathD^wt^ vs. cathD^-/-^; cathD^D33G^ | | <0.05* |
|  |  | cathD^-/-^; cathD^wt^ vs. cathD^-/-^; cathD^D75G^ | | <0.001*** |
|  |  | cathD^-/-^; cathD^wt^ vs. cathD^-/-^; cathD^E117G^ | | <0.01** |
|  |  | cathD^-/-^; cathD^wt^ vs. cathD^-/-^; 10xhis | | <0.01** |
